# Aqueous Phase Reforming over Platinum Catalysts on Doped Carbon Supports: Exploring Platinum–Heteroatom Interactions

**DOI:** 10.1021/acscatal.3c05385

**Published:** 2024-03-04

**Authors:** Monica Pazos Urrea, Simon Meilinger, Felix Herold, Jithin Gopakumar, Enrico Tusini, Andrea De Giacinto, Anna Zimina, Jan-Dierk Grunwaldt, De Chen, Magnus Rønning

**Affiliations:** † Department of Chemical Engineering, Norwegian University of Science and Technology, 7491 Trondheim, Norway; ‡ Institute for Chemical Technology and Polymer Chemistry, Karlsruhe Institute of Technology, Engesserstraße 20, 76131 Karlsruhe, Germany; § Institute of Catalysis Research and Technology, Karlsruhe Institute of Technology, Hermann-von-Helmholtz Platz 1, 76344 Eggenstein-Leopoldshafen, Germany

**Keywords:** Aqueous phase reforming, carbon nanofibers, heteroatom doping, hydrogen production, X-ray absorption spectroscopy

## Abstract

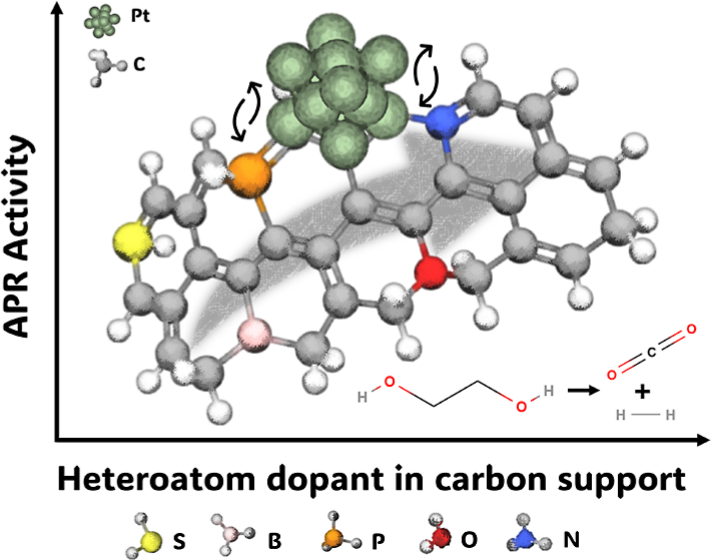

A series of platinum catalysts supported on carbon nanofibers with various heteroatom dopings were synthesized to investigate the effect of the local platinum environment on the catalytic activity and selectivity in aqueous phase reforming (APR) of ethylene glycol (EG). Typical carbon dopants such as oxygen, nitrogen, sulfur, phosphorus, and boron were chosen based on their ability to bring acidic or basic functional groups to the carbon surface. In situ X-ray absorption spectroscopy (XAS) was used to identify the platinum oxidation state and platinum species formed during APR of EG through multivariate curve resolution alternating least-squares analysis, observing differences in activity, selectivity, and platinum local environment among the catalysts. The platinum-based catalyst on the nitrogen-doped carbon support demonstrated the most favorable properties for H_2_ production due to high Pt dispersion and basicity (H_2_ site time yield 22.7 h^–1^). Direct Pt–N–O coordination was identified by XAS in this catalyst. The sulfur-doped catalyst presented Pt–S contributions with the lowest EG conversion rate and minimal production of the gas phase components. Boron and phosphorus-doped catalysts showed moderate activity, which was affected by low platinum dispersion on the carbon support. The phosphorus-doped catalyst showed preferential selectivity to alcohols in the liquid phase, associated with the presence of acid sites and Pt–P contributions observed under APR conditions.

## 1 Introduction

The implementation of renewable hydrogen is regarded as a crucial factor in the transition toward more sustainable energy systems. Currently, there is growing interest in exploring abundant and potentially carbon-neutral alternatives such as water and biomass to replace traditional primary sources, such as natural gas, for hydrogen production. Considering this perspective, aqueous phase reforming (APR) emerges as a prospect to utilize low-value mixed polyol streams to produce a gas mixture rich in hydrogen.^1^ Hence, APR can be exploited to valorize wastewater streams containing oxygenates from biomass processing to reduce the amount of waste generated and simultaneously obtain valuable products.

The APR process is typically carried out at moderate temperatures (220–270 °C) and pressures ranging from 20 to 60 bar.^2^ During this process, alcohols and sugars are converted to CO and H_2_, while CO is further converted to H_2_, and CO_2_ facilitated by the thermodynamically favorable conditions for the water-gas shift (WGS) reaction. However, the selectivity toward hydrogen is challenged by side reactions, including the formation of alkanes through methanation promoted at APR conditions and dehydration/hydrogenation reactions producing alcohols and organic acids at the expense of H_2_.^1,3^

Extensive research has been conducted on Pt as the catalyst of choice for the APR due to its favorable combination of high activity and selectivity toward H_2_ production.^4−6^ However, the product distribution is heavily influenced by the interactions between reactants as well as intermediates and the active sites, the nature of the support and their interphase, playing a fundamental role in determining the different steps taking place in the APR mechanism.^7−9^ Thus, the hydrogen selectivity can be tuned by altering the nature of the catalytically active metal site.^10,11^

In carbon-based catalysts, the substitution of carbon atoms within the carbon framework with elements that are nearby in the periodic table, such as O, N, B, P and S, is a common approach to modify the catalyst performance due to the effects emerging from the cooperative interactions between the metal and the modified support.^12,13^ The differences in the size, bond length, and electronegativity of the heteroatom can cause defects in the carbon surface. These defects can lead to localized charge accumulation depending on the electron affinity of the elements. As a result, it may significantly impact the adsorption/desorption properties on the catalyst surface which in turn will be reflected in the catalytic activity.^14−16,18^ Furthermore, smaller ensembles of metal particles can be formed by coordinating metal atoms with the dopants, acting as anchoring sites, to enhance the dispersion of the active phase on the surface.^19^

Carbon supports containing oxygen functional groups are effective in providing strong binding sites for metal nanoparticles. They are commonly used to enhance the hydrophilicity of carbon supports, which facilitates the diffusion of reactants on the catalyst surface.^20^ Moreover, an increase in surface basicity of the support has been observed when nitrogen is used to functionalize the carbon structure, which turned out to be beneficial in APR of polyols.^18,21^ Gogoi et al.^22^ studied APR of glycerol and observed that incorporating nitrogen into mesoporous carbon, along with ruthenium, resulted in increased dispersion and catalyst stability. This improvement in activity was attributed to enhanced electronic interactions between nitrogen and the d-orbitals of the metal due to a decrease in electron density around the Ru atoms.

The investigation of sulfur doping in the context of APR has not been explored, but since S mostly known as a catalyst poison,^23^ it can be used to suppress certain side reactions to influence the selectivity. For example, Auer et al.^24^ used sulfur to block defective metal sites on Pt/Al_2_O_3_ and promote the overall hydrogenation performance, by lowering the adsorption strength of the products facilitating their desorption. Likewise, phosphorus functional groups are known to provide hydrophilic acidic carbon surfaces due to the tendency of P to bind to oxygen. Thus, these kind of supports are exploited in acidic catalytic reactions such as dehydration, dehydrogenation, and oxidation.^25^ Meanwhile, boron-doped supports favor high dispersion of the metal particles and lower the adsorption strength of CO on the Pt surface, as observed by Wang et al.,^26^ resulting in favorable conditions for electrocatalytic applications.

Accordingly, these elements provide doped carbon supports with a wide range of properties that can be exploited for catalytic applications. They may be an alternative for seeking cost-efficient catalysts in the APR, which would lower the amount of costly noble-metal-based catalysts and improve their efficiency. Esteve-Adell et al.^27^ opted to study doped graphenes (N, B, and P) as a metal-free catalyst to promote the APR of glycerol. Boron-doped graphene was found to have the highest catalytic activity due to frustrated Lewis acid–base pairs formed in combination with negatively charged oxygen groups on the graphene surface. However, despite having the highest activity, B-doped graphene produced hydrogen only at an order of magnitude lower rate than that achieved by a Pt-based catalyst.

Aligned with these concepts, this study aims to explore the effects caused by heteroatoms present in the carbon nanofibers on the catalytic activity and their contribution to the overall performance of Pt-based catalysts in the APR of ethylene glycol (EG). Oxygen, nitrogen, sulfur, phosphorus, and boron were used to functionalize the carbon nanofiber surface, resulting in catalysts with diverse properties that strongly influence the APR activity.

## 2 Experimental Section

### 2.1 Synthesis of Carbon Nanofibers

A detailed description of the synthesis of platelet carbon nanofibers can be found elsewhere.^28^ Briefly, the carbon nanofibers were synthesized by chemical vapor deposition in a tubular quartz reactor using an Fe_3_O_4_ catalyst and CO/H_2_ as gas precursors. The Fe catalyst was synthesized by coprecipitation under N_2_ atmosphere, following the method described by Kang et al.^29^ The metal precursors used were FeCl_2_ (Sigma-Aldrich, ≤100%) and FeCl_3_ (Sigma-Aldrich, ≤100%), while NaOH (Sigma-Aldrich, ≤100%) was used as a precipitating agent. Approximately 100 mg of the catalyst was reduced at 600 °C for 6 h in 25% H_2_/Ar. Then, the system was flushed with Ar for 0.5 h, followed by the introduction of a synthesis gas mixture of CO/H_2_ in a volume ratio of 4:1 (total flow 62.5 mL/min) for 46 h at 600 °C. After the synthesis, the formed carbon was cooled to room temperature in flowing argon.

#### 2.1.1 HNO_3_ Treatment of CNF

For purification and to introduce oxygen functional groups to the carbon nanofiber surface, the CNFs were exposed to an acid treatment at 120 °C for 24 h in concentrated nitric acid (Sigma-Aldrich, 65 wt %), after which the suspension was filtered (Whatman, grade 589/2 filter paper) and thoroughly washed with deionized water until it reached a pH of 7. This purification procedure was carried out three times. After the last purification step, the carbon nanofibers were dried at 120 °C overnight in static air. The oxidized carbon nanofibers are referred to as CNF-ox.

#### 2.1.2 Heat Treatment of CNF

The CNF-ox were subjected to a 2 h heat treatment in argon atmosphere at 700 °C. This process took place within a quartz reactor located inside a vertical tubular furnace. The main objective of this heat treatment was to remove unstable oxygen functional groups from the carbon surface that had been introduced during acid treatment. The heat-treated CNF is denoted as CNF-HT.

#### 2.1.3 Heteroatom Doping of Carbon Nanofibers

The functionalization of the carbon nanofibers either by nitrogen, sulfur, or phosphorus was conducted following the gasification-assisted heteroatom doping method (GAHD) proposed by Herold et al.^30^ The method involves inducing carbon surface defects using a gasification agent in the presence of a gaseous heteroatom source at high temperatures. Hence, the heteroatoms occupy the newly formed sites, resulting from carbon gasification.

In short, the carrier gas is saturated with a heteroatom precursor solution and fed into a quartz reactor containing CNF-ox through a saturator located upstream from a tubular vertical furnace at a desired temperature and reaction time. Throughout the heating and cooling stages of the treatment, the carrier gas was supplied directly into the reactor, bypassing the saturator. 350 mg of CNF-ox was used to conduct each doping experiment. The procedure was repeated to generate sufficient material for the catalyst preparation. Figure S1 depicts a schematic representation of the setup employed for heteroatom functionalization of the CNF-ox.

##### 2.1.3.1 Synthesis of N-Doped Carbon Nanofibers

The reactor was loaded with CNF-ox and heated to 875 °C at a rate of 10 °C/min in a N_2_ atmosphere with a flow of 250 mL/min. To saturate the nitrogen stream, it was passed through a sparger that contained a solution of ethylene diamine (Sigma-Aldrich, ≥99.5%) and water in a molar ratio of 1:1.5, before being fed into the reactor. After an hour of exposure time, the system was cooled in a N_2_ atmosphere.

##### 2.1.3.2 Synthesis of P-Doped Carbon Nanofibers

To dope the surface of CNF-ox with phosphorus, the reactor was heated to 825 °C at a rate of 10 °C/min in a H_2_ flow of 50 mL/min. The carrier gas flow was passed through a sparger filled with trimethyl phosphite (Sigma-Aldrich, ≥99%) and fed into the reactor for 3 h. The inlet gas was switched to 100 mL/min N_2_ for the cooling phase. Once the temperature was below 80 °C, synthetic air was supplied to oxidize possibly formed white phosphorus overnight.

##### 2.1.3.3 Synthesis of S-Doped Carbon Nanofibers

For sulfur treatment, the reactor was heated at 10 °C/min in a mixed carrier gas of 90 mL/min N_2_ and 10 mL/min CO_2_. At 825 °C, an additional 2 mL/min N_2_ flow was passed through the saturator containing pure carbon disulfide (Sigma-Aldrich, ≥99.9%) and directed toward the reactor for an exposure time of 11 min. After the reaction time, the reactor was cooled in a N_2_ flow of 90 mL/min. During the experiment, the saturator containing the sulfur source was cooled with a mixture of ice and NaCl (10:1 g/g) due to the high volatility of CS_2_. As a final step, the S-doped CNF was exposed to H_2_ at 450 °C for 30 min with a heating rate of 10 °C/min to remove sulfur-containing groups on the surface of the CNF that are unstable in a H_2_ atmosphere.

##### 2.1.3.4 Synthesis of B-Doped Carbon Nanofibers

The functionalization of the carbon nanofibers by boron was carried out following the wet impregnation method proposed by Chiang et al.^31^ Briefly, aiming at a boron loading of 3 wt %, 2.5 g of CNF-ox was suspended in 250 mL of DI water in addition to 0.23 g of B_2_O_3_ (Sigma-Aldrich, ≤100%) in a 500 mL round-bottom flask. After 25 min in an ultrasonic bath, the suspension was heated to 80 °C in an oil bath for 2 h and mixed with a magnetic stirrer at 500 rpm. Subsequently, the mixture was dried in an oven for 24 h at 60 °C, followed by a heat treatment at 1000 °C for 8 h in the argon atmosphere using a vertical tubular furnace.

A washing procedure was necessary to remove the excess physisorbed boron oxide in the synthesized B-CNF. Hence, the B-CNF was suspended in DI water for 25 min in an ultrasonic bath, followed by a 2 h exposure to 80 °C in an oil bath with magnetic agitation. Subsequently, the hot suspension was filtered and dried for 14 h at 60 °C.

### 2.2 Catalyst Synthesis

The catalysts were synthesized by incipient wetness impregnation with a solution of chloroplatinic acid hexahydrate (H_2_PtCl_6_·6H_2_O, Sigma-Aldrich, >37.5% Pt) in acetone (Sigma-Aldrich, ≥99%). The solution was prepared to achieve a Pt loading of 3 wt %. The support material was mixed dropwise with the Pt solution and dried in air for 2 h at room temperature, followed by a drying step for 12 h at 80 °C.

The prepared catalysts underwent a heat treatment in N_2_ (100 mL/min) at 320 °C with a heating rate of 3 °C/min for 2 h. This was followed by reduction at 300 °C under 100 mL/min 10% H_2_/N_2_ flow for 1 h prior to APR.

### 2.3 Catalyst Characterization

N_2_ physisorption was employed to measure the textural properties of the carbon supports using a Micromeritics Tristar 3000 instrument at a temperature of −196 °C. Approximately 100 mg of the samples was subjected to overnight degassing at 200 °C before the analysis. The specific surface area was determined by the Brunauer–Emmett–Teller (BET) method^32^ using 11 points in the *p*/*p*0 = 0.05–0.3 range.

CO chemisorption was carried out on a Micromeritics ASAP 2020 instrument. Approximately 100 mg of the sample was subjected to in situ reduction for 1 h in pure H_2_ at 300 °C with a heating rate of 5 °C/min. Following the reduction, the system was purged with helium at 120 °C for 30 min before being cooled to 35 °C and evacuated for the chemisorption analysis. Pt dispersion was evaluated assuming a Pt/CO adsorption stoichiometry of 1.^33^ The estimation of the particle size (d) in nanometer was conducted under the assumption of spherical geometry. The metal dispersion (*D*) was estimated according to eq 1, using the adsorption stoichiometry of 1 (*F*), monolayer of CO uptake (*n*_co_), and molar mass of the metal (*M*_Pt_) and mass fraction of the metal on the catalyst sample (*w*_Pt_).
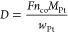
1

Raman spectroscopy was performed using a Horiba Jobin Yvon LabRAM HR800 Raman microscope using a HeNe laser operating at a wavelength of 633 nm. The spectra were collected within the range 750 to 3250 cm^–1^, employing an acquisition time of 30 s and averaging the results from five accumulations. The data fitting and information about the G and D band were carried out according to Mallet-Ladeira et al.^34^ Five distinct locations on the surface of each sample were examined and averaged.

X-ray photoelectron spectroscopy (XPS) was performed on a Kratos Analytical Axis Ultra DLD spectrometer using monochromatic Al Kα radiation (1486.6 eV) operating the anode at 10 kV with an aperture of 700 μm × 300 μm. Survey scans were recorded using a pass energy of 160 eV, and high-resolution spectra were acquired using a pass energy of 20 eV. The energy axis was calibrated to the C 1s contribution of sp^2^ carbon at 284.6 eV. The peaks were deconvoluted after Shirley background subtraction^35^ using linear combinations of Gaussian and Lorentzian functions. The full set of band assignments as well as fitting parameters are listed in Table S1. The platinum binding energy of 71.7 eV,^30^ related to Pt supported on carbon nanofibers with a low degree of functionalization, was used to compare the XPS shifts of the binding energy of Pt supported on the heteroatom-doped carbon nanofibers. The XPS analysis was performed on the reduced catalysts before and after APR. This means that the catalysts were exposed to air when transported to the XPS chamber. Surface oxidation of the platinum nanoparticles is assumed after exposure to air.

Temperature-programmed desorption of ammonia experiments (NH_3_-TPD) were performed in a catalyst analyzer BELCAT II (Microtrac-Bel Japan Inc.) equipped with a thermal conductivity detector (TCD). To obtain the NH_3_-TPD profiles, a pretreatment procedure was performed on 70 mg of the sample. First, the sample was heated in a He atmosphere at 110 °C for 60 min and then cooled to 40 °C. Next, it was saturated with a 5% NH_3_/He mixture for 1 h at a flow rate of 30 mL/min, followed by a 1.5 h purge in He. Finally, the temperature was raised to 610 °C at a heating rate of 10 °C/min under flowing He (30 mL/min).

Zeta potential was measured on a Malvern Nano-ZS Zetasizer (Malvern Instruments, UK). The electrophoretic mobility was measured by laser Doppler velocimetry. The zeta potential was determined by the Smoluchowski approximation.^36^ Prior to the analysis, 20 mg of the catalyst was dispersed in 50 mL of DI water by 30 min of ultrasonication, including measurement of pH of the dispersion (in the range of 4.5–5.5).

Scanning transmission electron microscopy (STEM) images were obtained on a Hitachi SU9000 electron microscope operating at an accelerating voltage of 30 kV. Energy-dispersive X-ray spectroscopy (EDS) maps were acquired at an accelerating voltage of 30 kV using an Oxford Ultim Extreme 100 mm^2^ windowless detector. Samples were prepared by ultrasonic dispersion in *n*-hexane followed by drop-casting on carbon-coated copper grids. Particle size distributions were determined using ImageJ software, including 300–600 particles.

Microwave plasma atomic emission spectroscopy (MP-AES) was used to determine the metal loading of the catalyst with an Agilent 4210 MP-AES optical emission spectrometer. The samples were prepared through microwave-assisted digestion by utilizing a Berghof Speedwave XPERT instrument. For the digestion, the catalyst was dissolved in 10 mL mixture of HCl and HNO_3_ (1:4, vol/vol). The process comprised two steps; initially heating up to 170 °C and maintaining the temperature for 10 min, followed by a temperature increase to 210 °C for 20 min with a power of 2 × 800 W. In preparation for the analysis, the samples were placed into a 100 mL volumetric flask, and the volume was adjusted using Millipore Milli-Q water. A syringe filter with a pore size of 0.2 μm was used to filter the samples.

In situ powder XAS-XRD experiments were carried out at BM31 of the Swiss-Norwegian beamlines (SNBL) at the European Synchrotron Radiation Facility (ESRF), France. A dedicated setup with mass flow controllers (Bronkhorst), back pressure regulator (Bronkhorst), and HPLC pump (Flusys GmbH, WADose-Lite HP) was used to feed the desired components to the system; N_2_, H_2_, and 6 wt % aqueous solution of EG. A detailed setup scheme can be seen in Figure S2. Catalyst samples (8 mg, 120–200 μm sieve fraction) were loaded into a quartz capillary tube with 2.0 mm internal diameter and 0.02 mm wall thickness. Quartz wool plugs and Kapton polyamide tube pieces (MicroLumen) were placed at either end of the catalyst bed (bed length of 1 cm) to keep the catalyst in place during the reaction. The capillary reactor was mounted in a custom cell and exposed to X-rays, while the capillary temperature was regulated using a hot air blower. X-ray diffractograms were collected with a Pilatus3 2 M detector (Dectris) using monochromatic radiation (λ = 0.255 Å). The instrumental peak broadening, wavelength calibration, and detector distance corrections were performed using a NIST 660a LaB_6_ standard. X-ray absorption spectra were recorded at the Pt L_3_ edge (11.564 keV). Data collection was carried out in the transmission mode. XANES profiles were recorded during reduction of the catalyst (300 °C, 5% H_2_/He flow, 1 h) and during APR of EG at 225 °C, 30 bar and 9 h^–1^ WHSV (0.02 mL/min) for 2 h. Additionally, in situ X-ray absorption spectroscopy (XAS) experiments were conducted for the Pt/CNF-HT sample at the CAT-ACT beamline at the KIT Light Source.^37^ The experimental setup was specifically designed for high-pressure applications and featured a high-pressure cell that facilitated XAS and XRD measurements (Figure S3). Detailed information about the setup can be found in the publication by Loewert et al.^38^

EXAFS were measured before and after reduction and APR at 50 °C to analyze the local environment of Pt for fresh, reduced, and spent catalysts. Platinum standards, EXAFS of platinum foil (Pt^0^) and PtO_2_ (Pt^4+^) were measured ex situ in the transmission mode. The EXAFS data were analyzed using Athena and Artemis components of the Demeter software package.^39^ Data analysis of the CNF-HT, N-CNF, and S-CNF-supported platinum catalysts was performed by multiple shell fitting in *k* space in k^2^-weighting. However, EXAFS fitting of the data from the P-CNF and B-CNF-supported platinum catalysts in *k* space did not lead to a satisfactory fit. Thus, for these samples, the data were fitted in *R* space in the range 3 < *k* < 12 Å^–2^, 1.1 < *R* < 4 Å. The number of floating parameters used in the analyses satisfied the Nyquist criterion.^40,41^

The multivariate curve resolution alternating least-squares (MCR-ALS)^42,43^ package in Python (3.10) was used for analyzing the collected XANES TPR and APR data for the catalysts.^44^ The XAS standards for PtN, PtN_2_, PtB, PtP, PtP_2_, PtO, PtO_2_, PtS, PtS_2_, and Pt were obtained from the Materials Project database.^45−48^ Prior to MCR analysis, the XANES TPR and APR data for all catalysts and all standards from the materials project were preprocessed using a normalization function between 11.56 and 11.6 keV.^49^ The extracted normalized XANES data were subjected to MCR-ALS with a precise set of pretreated standards from the materials project to extract a contribution plot with minimal error.

### 2.4 Aqueous Phase Reforming of Ethylene Glycol

APR of EG was carried out in a 160 mL stainless-steel mini bench batch reactor (Parr 4592 model) equipped with a magnetic driver stirrer and PID temperature controller (Parr Instruments Co., USA). The reactor vessel was loaded with 150 mg of the prereduced catalyst and 30 mL of aqueous EG solution with a concentration of 6% wt. After the reactor was sealed, it underwent a leak test at pressures exceeding 120 bar. Subsequently, the reactor was purged with nitrogen and pressurized to an initial pressure of 26 bar, which was utilized as an internal standard to accurately quantify the products present in the gas phase. The mixture was stirred and heated to 250 °C for 25 min. After 2 h, the reaction was quenched to room temperature using an ice bath.

The gaseous products were gathered in a gas sampling bag (Supelco) and analyzed by gas chromatography (Agilent 7820A, equipped with a TCD detector and flame ionization detector FID). The columns used for separation of the permanent gases were Agilent Porapak-Q GS-Q and CP-Molsieve 5 Å, while J&W HP-PLOT Al_2_O_3_ KCl column was used for analysis of the light hydrocarbons.

The liquid products were collected, filtered using a 0.2 mm PTFE filter, and analyzed by high-performance liquid chromatography (1260 Infinity II LC System, Agilent Technologies, equipped with a refractive index detector). A Hi-Plex H ion exclusion column (300 mm × 7.7 mm) was used to separate the liquid products. The separation process was carried out at a flow rate of 0.6 mL/min and a temperature of 60 °C, using a 5 mM sulfuric acid solution. External standard calibration, including expected reaction products, was employed.

The spent catalysts were retrieved through filtration, rinsed with deionized (DI) water, and dried at 60 °C overnight. Table S2 displays a summary of the equations and parameters used to evaluate the APR catalyst performance.

## 3 Results and Discussion

### 3.1 Aqueous Phase Reforming of EG

APR of EG was conducted in a batch reactor to assess the catalyst performance with a particular focus on the impact of the doped support. A blank experiment in the absence of a catalyst and control experiments with doped-CNF supports were conducted. As expected from previous studies using doped graphene in APR of glycerol,^27^ ethanol and small traces of H_2_ and methane were detected as products (Figure S4).

**Figure 1:**
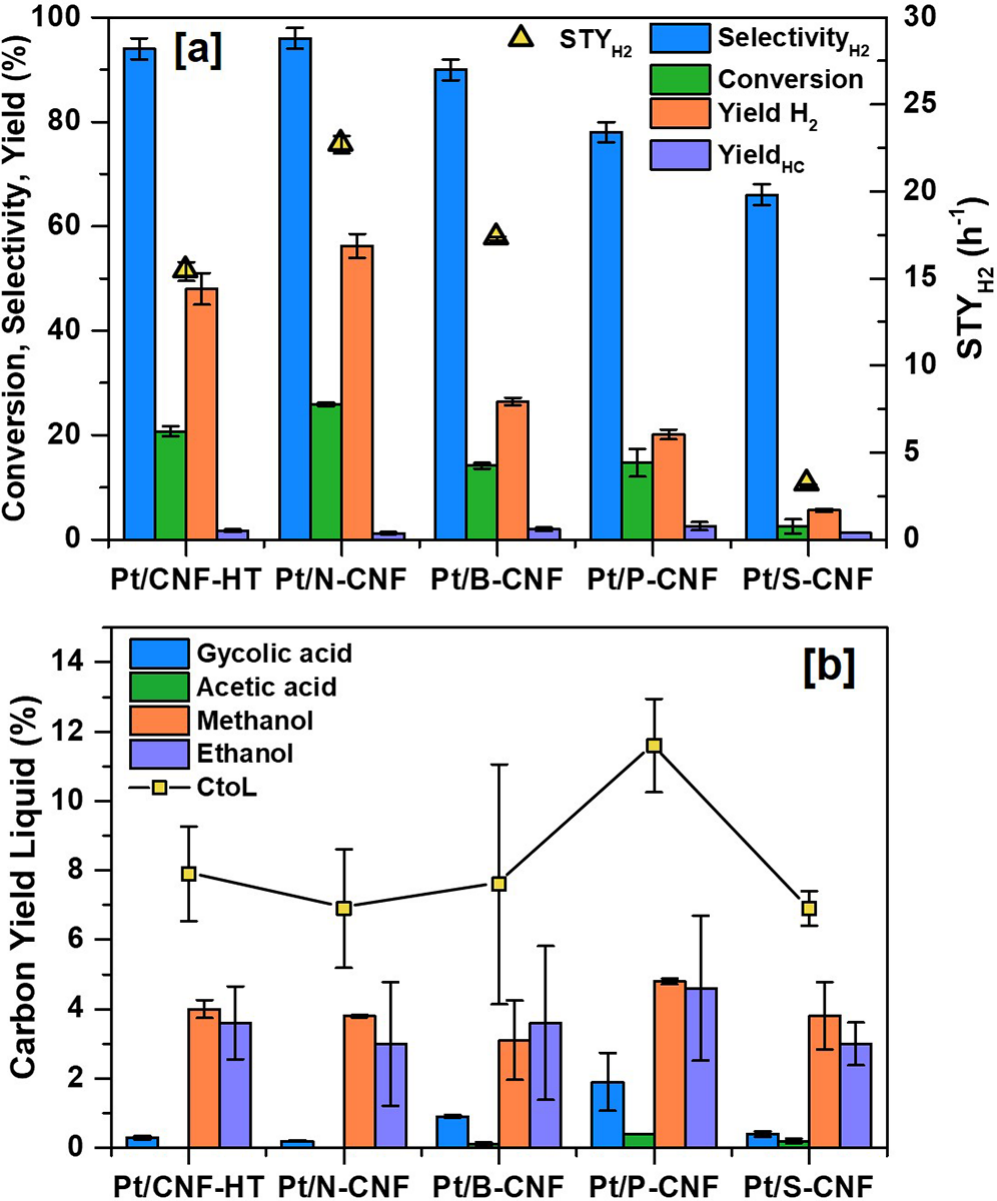
APR of 6 wt % ethylene glycol (EG) aqueous solution at 250 °C and 26 bar initial pressure in the batch reactor. (a) Catalytic conversion of EG, hydrogen selectivity, hydrogen and hydrocarbon yield, and H_2_ site time yield. (b) Carbon yield to main liquid products and total conversion of carbon to liquids (CtoL). The error bars represent the standard deviation in the experiments. The parameters are calculated based on Table S2.

The results showed that Pt/N-CNF had the highest EG conversion among the catalysts, followed by Pt/CNF-HT, Pt/P-CNF, Pt/B-CNF, and Pt/S-CNF in the descending order (Figure 1a). In general, the gas phase consisted mainly of H_2_ and CO_2_, with small portions of methane (<3.4% mol) and ethane (<2.6% mol). Traces of higher alkanes, ethene, and CO were also observed (Table S3).

Detected products in the liquid phase represented less than 15% of the total carbon feed. Measurements of the liquid effluent of the various experiments yielded final pH values of around 3–4, indicating the presence of acidic species, as observed in the liquid product distribution. The main products identified in the liquid phase include glycolic acid, acetic acid, ethanol, and methanol (Figure 1b), with a larger yield of alcohols relative to organic acids. All measurements have been confirmed to have a carbon balance accuracy ranging from 88 to 100%.

APR results using Pt-based catalysts supported on heteroatom-doped CNF (Figure 1) showed that the reforming ratio of H_2_ to CO_2_ was close to the theoretical values, which during APR reaction of EG is expected to be 2.5.^50^ This observation suggests that the series of catalysts evaluated did not significantly promote the consumption of H_2_ through side reactions except for Pt/P-CNF with a reforming ratio of 2.2. The reason for a lower reforming ratio for this catalyst could be the hydrogenation of unsaturated intermediates, leading to consumption of the produced H_2_,^51^ with larger fractions of hydrocarbons and liquid byproducts in the chemical species formed (Figure 1).

It has been observed that the apparent selectivity toward reforming (indicated by the presence of H_2_ and CO_2_) decreases as the particle size of platinum increases. Simultaneously, the formation of light alkanes and the yield to liquid products tend to increase with the platinum particle size. This trend suggests that Pt plays a primary role in hydrogen production through C–C cleavage, which is consistent with observations in previous research.^2,51^

Due to several influencing parameters, such as pressure, gas-to-liquid ratio, and initial concentration, comparing the current findings with previous results of APR of EG presents a challenging task. This is particularly difficult in batch reaction conditions, where the accumulation of the products may negatively affect the activity and stability of the catalyst, such as organic acids or CO in the gas phase, including high autogenous pressures in the reaction.^52^ However, activity reported by van Haasterecht et al.^53^ for Pt-based catalyst supported on oxidized carbon nanofibers, achieved an H_2_ site time yield (STY) of 0.26 min^–1^ at 230 °C, while the most active catalyst from the present series (Pt/N-CNF) resulted in an H_2_ STY of 45.4 min^–1^ at a higher temperature of 250 °C.

### 3.2 Catalyst Characterization

#### 3.2.1 Initial Characterization of the Catalyst

The surface area of CNF obtained by the BET method varies from 159 m^2^/g for CNF-HT to 71 m^2^/g for P-CNF, decreasing in the following order: CNF-HT ∼ S-CNF > N-CNF > B-CNF > P-CNF, as can be seen in Table 1. The heat treatment of the CNF-ox allowed the formation of defects on the surface by decomposition of oxygen containing groups to CO and CO_2_ at 700 °C, resulting in a higher-surface area.^54^ Similarly, during S doping, a comparatively high carbon gasification rate may lead to an increase in the surface area.^55^ The textural changes of N-CNF, B-CNF, and P-CNF can be referred to a decrease in the total pore volume, as shown by lower N_2_ uptake in the adsorption isotherms (Figure S5). It is possible that the decrease in surface area of N-CNF is due to the presence of water in the doping treatment, which may have caused decomposition of amorphous carbon on the CNF. However, in the case of P-CNF, it is likely that the significant decrease in surface area is a result of the deposition of P species on top of the carbon surface.^30^ As observed in previous studies by Song et al.^15^ on phosphorus-doped ordered mesoporous carbon, an excessive amount of the phosphorus content resulted in a reduction of micropores, consequently leading to a decrease in the BET surface area.

**Table 1:** Pt Loading, Dispersion, and Average Particle Size of the Reduced and Spent Catalyst after APR of 6 wt % EG at 250 °C and 26 bar Initial Pressure

catalyst	BET surface area (m^2^/g)	Pt loading^a^ (±0.1 wt %)		Pt dispersion^b^ (±1%)		CO uptake^b^ (μmol/g STP)		Av. Pt particle size^b^ (nm)		Av. Pt particle size^c^ (nm)	
		red	spent	red	spent	red	spent	red	spent	red	spent
Pt/CNF-HT	159	2.6	2.7	47	28	62.5	39.2	2	4	1.7 ± 1.5	2.6 ± 2.0
Pt/N-CNF	114	2.4	2.5	43	29	53.6	37.2	3	4	2.0 ± 1.1	1.9 ± 1.1
Pt/S-CNF	156	2.7	2.7	26	11	35.7	15.1	4	10	1.4 ± 0.7	1.9 ± 1.5
Pt/P-CNF	71	2.8	2.9							2.5 ± 2.3	2.2 ± 1.1
Pt/B-CNF	113	3.2	3.2	19	9	31.3	15.4	6	12	2.0 ± 2.4	2.4 ± 2.7

a Determined by MP-AES.

b Estimated from CO chemisorption.

c Estimated by particle size distribution and standard deviation obtained from the TEM images (Figure S8).

The graphitization level of the carbon support was assessed by analyzing the relative intensity of the G band compared to the D band, and the G band position by Raman spectroscopy.^34^ The Raman spectra of the CNF supports, accompanied by their respective calculated *I*_D_/*I*_G_ intensity ratio, are presented in Figure S6. In this study, the samples displayed *I*_G_/*I*_D_ values close to 2, indicating the presence of graphitic carbon along with a high concentration of edge sites,^34^ which is characteristic of platelet CNF. Notably, no significant variations were observed with the introduction of the different heteroatoms that may induce further structural distortion, holes, and unoccupied carbon sites, resulting in an increase in the ratio.^56^

Similar observations are derived from minimal shifts in the G band (Figure S7), which is typically sensitive to chemical doping.^57^ The G band position can experience a downshift when electron-donating heteroatoms, such as N, S, and P, are introduced, while an upshift occurs when electron-withdrawing heteroatoms (such as B) are present.^17,58^ For the catalyst supports studied, the structural defects in the bulk CNF might predominate over the alterations caused by substituting a carbon site with a heteroatom on the surface, hindering the identification of differences in descriptors such as *I*_D_/*I*_G_ and shifts in the G band. Hence, the doping process had a reduced impact on the overall structure of the carbon nanofibers compared to CNF-HT, as evidenced by previous studies.^30^ Despite alterations in the carbon texture, the fundamental structure of the carbon nanofibers remained largely unaltered with a heteroatom doping level of about 2% (Figure 3f).

An advantage of utilizing carbon materials doped with heteroatoms is the feasibility of improving the dispersion of metal nanoparticles on the carbon support and preventing their agglomeration. The heteroatoms can serve as anchoring points for the particles resulting in enhanced stability.^12,14,59^ In this study, the dispersion and particle size exhibited notable differences depending on the functionalization of the CNF support. Table 1 displays the Pt dispersion, Pt particle size, and metal loading of the reduced and spent catalysts. Among the catalyst series, Pt/CNF-HT and Pt/N-CNF exhibit the highest Pt dispersions at 47 and 43%, respectively. Conversely, Pt/S-CNF has a low Pt dispersion value of 26%, followed by Pt/B-CNF at 19%.

**Figure 2:**
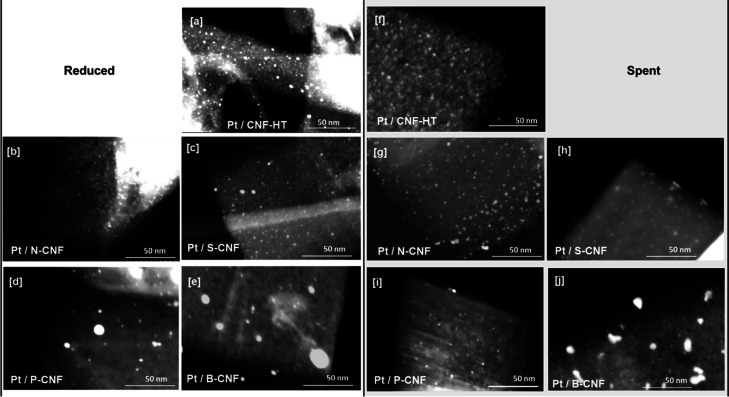
STEM images of the reduced catalyst (a) Pt/CNF-HT, (b) Pt/N-CNF, (c) Pt/S-CNF, (d) Pt/P-CNF, and (e) Pt/B-CNF. STEM images of the spent catalyst (f) Pt/CNF-HT, (g) Pt/N-CNF, (h) Pt/S-CNF, (i) Pt/P-CNF, and (j) Pt/B-CNF after APR of 6 wt % of EG at 250 °C and 26 bar initial pressure.

Regarding Pt/P-CNF, it was observed that the CO chemisorption was strongly inhibited in the presence of phosphorus. Ding et al.^60^ suggested that the incorporation of P atoms into the carbon structure lowered the adsorption strength of CO on Pt–Ni, facilitating the removal of CO from the surface of the catalyst. Similarly, Song et al.^15^ noted that the inclusion of phosphates in carbon structures enhanced stability and CO tolerance in electrocatalytic applications when compared to nondoped catalysts. Based on these previous observations, low CO adsorption strength in the presence of P is a viable explanation for the low CO uptake on Pt/P-CNF. Note that the CO uptake was close to zero during the chemisorption analysis even though dispersed Pt nanoparticles could be observed in the STEM images (Figure 2d).

The spatial elemental distribution of the catalysts on the doped CNF was examined by STEM-EDS mapping (Figures S9–S13). They show that the carbon nanofibers contain heteroatoms uniformly distributed across the surface. The distribution of platinum varies depending on the sample, with a consistent and uniform dispersion in the CNF-HT, N-CNF, and S-CNF catalysts, while larger platinum particles are present in the P-CNF and B-CNF catalysts.

Analysis of the STEM images and CO chemisorption after the reaction demonstrated that the size of the nanoparticles increased during APR of EG (Table 1 and Figure 2). This indicates that the Pt nanoparticles on all the supports studied underwent some level of sintering under the reaction conditions. This effect was more pronounced in S- and B-doped catalysts, which presented particles that were 2.5–2 times larger, while the particle growth was less significant for CNF-HT and N-doped catalysts.

#### 3.2.2 XPS and Zeta Potential Characterization

**Figure 3:**
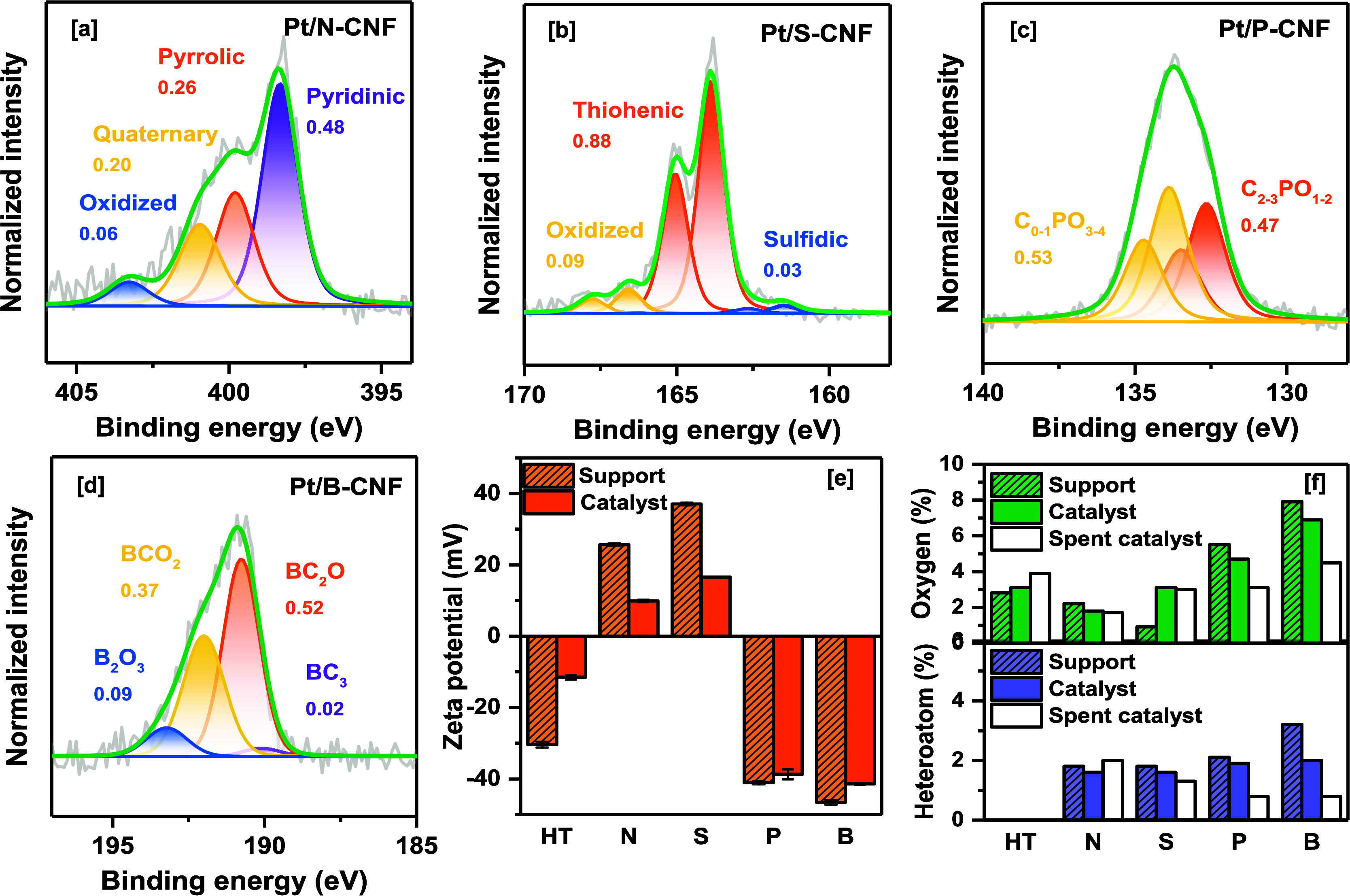
High-resolution XPS of (a) N 1s of Pt/N-CNF, (b) S 2p of Pt/S-CNF, (c) P 2p of Pt/P-CNF, and (d) B 1s of Pt/B-CNF reduced catalysts. (e) Zeta potential (mV) and (f) oxygen and heteroatom content (% at.) of the X-CNF supports and Pt-based reduced and spent catalysts.

Information about the presence and content of the related heteroatoms in the carbon surface and their percentage and distribution among the different molecular structures are determined by EDS mapping (Figures S9–S13) and XPS (Figures 3, S14, Table S4). In addition, zeta potential measurements related to the surface charge of the catalyst when dispersed in aqueous solution were analyzed (Figure 3e). In Pt/CNF-HT, the total oxygen content in the surface was approximately 3.1% at., displaying a zeta potential of −11.0 ± 0.5 mV. For Pt/N-CNF, the distribution of nitrogen species (1.6% at.) on the carbon surface was dominated by pyridinic N (48%), followed by pyrrolic N (26%), quaternary N (20%), and oxidized N (6%), resulting in a zeta potential of 10.1 ± 0.3 mV. Pt/S-CNF consisted of 3 S species (1.5% at.), mainly comprising thiophenes (89%), sulfoxides (9%), and sulfides (2%), with a zeta potential of 16.6 ± 0.1 mV. Pt/P-CNF exhibited both reduced P species (47%) and oxidized P species (53%). The measured zeta potential for Pt/P-CNF was −38.7 ± 1.3. Lastly, for Pt/B-CNF, the boron species (2% at. %) were distributed as BC_2_O (52%), BCO_2_ (37%), B_2_O_3_ (9%), and BC_3_ (2%), resulting in a zeta potential of −42.4 ± 0.2 mV.

The surface charge density is influenced not only by the quantity of functional groups but also by pH and the specific type of functional groups present on the carbon surface.^61,62^ For example, in the case of Pt/S-CNF, the most abundant species found on the carbon surface are thiophenes, a weakly acidic group. At low pH, weak acidic groups can acquire a positive charge due to proton transfer from the solution, or their dissociation can be suppressed, resulting in a positive zeta potential. Hence, the catalysts containing basic groups like pyrrolic, quaternary, and pyridinic nitrogen or weakly acidic groups (S, N) exhibited a positive zeta potential. On the other hand, samples with a higher concentration of acidic groups, such as hydroxyl (OH–) and carboxyl (COOH–) groups (HT, P, and B), displayed a negative zeta potential.

Additionally, it was observed that introducing functional groups significantly enhances the hydrophilicity of carbon nanofibers, leading to increased dispersibility in an aqueous environment, as noted in previous studies.^25,58,63^ In suspensions with zeta potential close to zero (±10) with a lower degree of functionalization, such as CNF-HT, aggregation and sedimentation of the CNF were observed in the aqueous solutions.

NH_3_-TPD (Figure S15) was conducted to evaluate the acidic properties of the catalysts. Multiple peaks in each profile suggest varying site strengths in the samples. The profiles for all catalysts display a peak around 100 °C, associated with weakly chemisorbed ammonia molecules. The amount of ammonia absorbed can be linked to the acidic level of the catalyst. Therefore, Pt/CNF-HT and Pt/N-CNF are less acidic catalysts compared to the other catalysts. Pt/P-CNF exhibited the highest desorption peak at 210 °C, most likely associated with P–OH, which is favorable for coordinating with NH_3_.^64^ Next in acidity, Pt/B-CNF has the highest desorption peak at 180 °C, including a shoulder at 255 °C, which may be related to oxidized boron species, as revealed by XPS analysis (Table S4). Finally, the Pt/S-CNF catalyst showed the highest desorption peak at 250 °C, indicating the presence of weak/medium acid sites on the surface. These sites may be attributed to the oxidized sulfur species indicated by XPS (Table S4).

**Figure 4:**
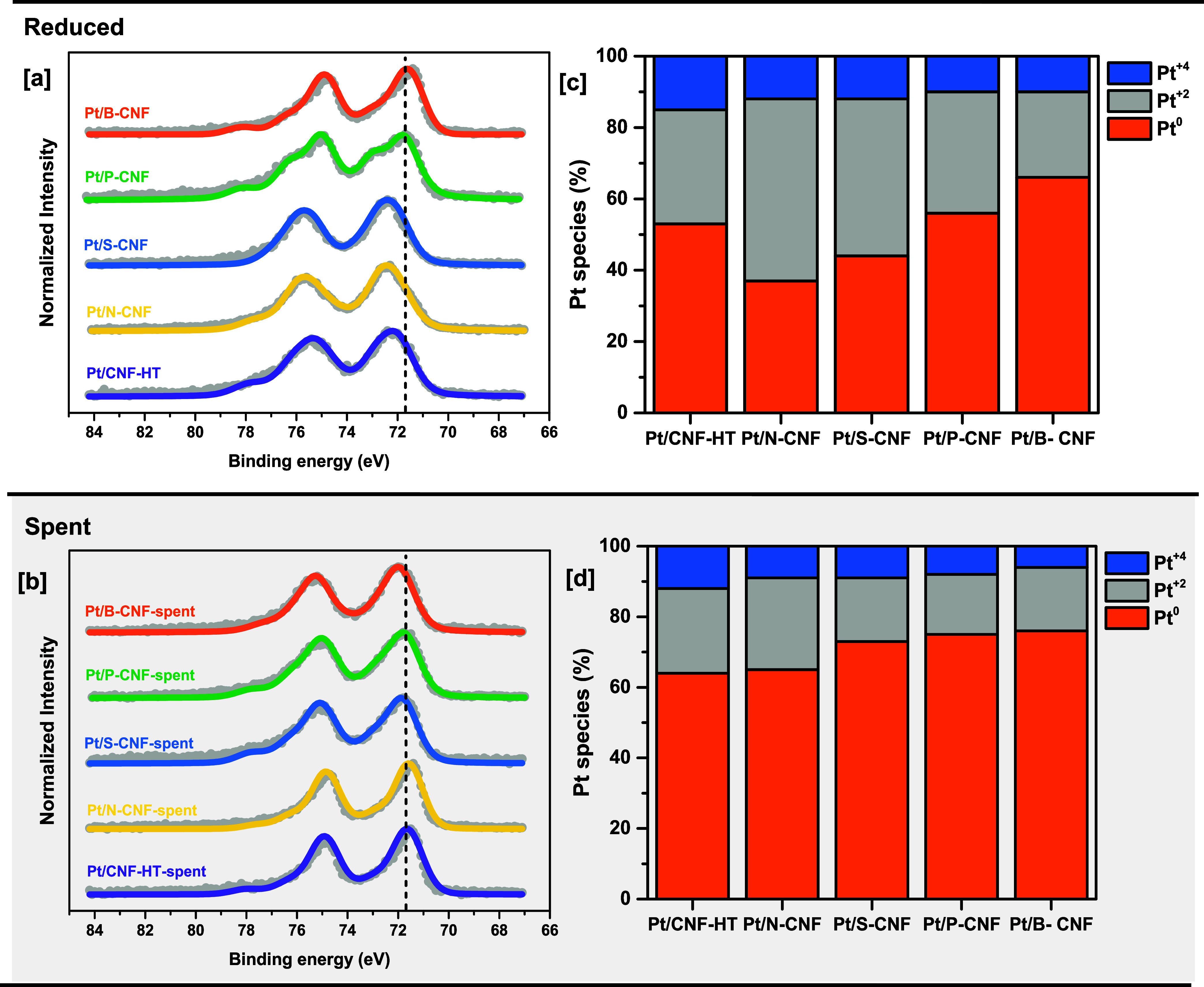
Normalized XPS Pt 4f spectra of Pt supported on heteroatom-doped CNF (a) after ex-situ reduction and (b) after APR. The vertical line at 71.7 eV indicates the binding energy of Pt on carbon nanofibers with a low degree of functionalization (c)^30^ Pt species fractions based on the deconvolution of the Pt 4f signal in HR-XPS for the reduced catalysts. (d) Pt species fractions based on the deconvolution of the Pt 4f signal in HR-XPS for the spent catalyst.

To investigate the relationship between the catalytic activity and the diverse interactions between the doped support and platinum, the electronic properties of platinum in the reduced and spent catalysts were evaluated by XPS. A shift toward higher binding energy of the core electron of Pt implies a decrease in the electronic charge density on the metal. Due to a transfer of charge from the metal to the support. A downshift in binding energy may indicate a reverse charge transfer.^65^ Thus, the presence of electron-withdrawing functional groups on the carbon structure could result in an increase in the binding energy, whereas electron-donating groups may lead to a decrease.^12,58,66,67^ The Pt/CNF-HT catalyst is functionalized to a certain extent with oxygen surface functional groups and contains approximately 3.1 at. % of oxygen. The presence of oxygen functional groups in the carbon surface may have an impact on the Pt binding energy similar to that of other heteroatoms. Based on the XPS spectra, it was observed that the Pt_4f_ signal exhibited a 0.3 eV positive shift compared to the binding energy of Pt^0^ (71.7 eV)^30^ for Pt/CNF-HT, Pt/N-CNF, and Pt/S-CNF, indicating the existence of an electronic interaction between Pt and the doped carbon supports (Figure 4, Table S5). However, the Pt/P-CNF and Pt/B-CNF showed no difference in comparison to the binding energy of Pt_4f_ in the metallic state.

Understanding the direct effects of the metal–support interactions can be challenging by the fact that the particle size of the active phase may also play a role.^68,69^ Thus, studies indicate that the impact of metal–support interactions varies based on the particle size and generally become more significant for metal nanoparticles smaller than 4 nm.^70^ As can be perceived from the particle size distribution from the STEM images (Figure S8), Pt/B-CNF and Pt/P-CNF are the samples with larger Pt particles among the catalyst studied, and thus the dopants on the carbon support may have less effect on the electronic structure of platinum.

By deconvoluting the Pt_4f_ signal (Figures S16 and S17), XPS analysis can provide insights into the composition of various Pt species present on the surface of the catalyst. For most catalysts, fully reduced Pt (Pt^0^) was observed to be the predominant Pt species. However, for Pt/N-CNF and Pt/S-CNF, only 37 and 44% of the Pt species, respectively, were determined to be in the metallic state. After exposing the catalyst to H_2_ at high temperatures, the low content of reduced platinum species could be explained by metal–support interactions and the exposure of the catalyst to air before conducting the ex-situ XPS measurements (rapid oxidation of especially small Pt clusters/nanoparticles). Additionally, in all catalysts, Pt^2+^ species were found to be more abundant than Pt^4+^ among the oxidized species, as can be seen in Figure 4.

After APR, it was observed that the content of functional groups incorporated into the surface of the carbon support decreased (Figure 3 f). This was particularly evident in samples containing P and B (∼40% loss), indicating that some species were not able to withstand the harsh hydrothermal conditions associated with APR. Heteroatoms that come in contact with the liquid phase might suffer oxidation. Thus, the dopant can migrate from the carbon surface into the aqueous solution when converted to water-soluble compounds (e.g., phosphate and boric acid).^71^ N-CNF and CNF-HT were stable and capable to maintain their heteroatom content throughout the reaction.

All spent catalysts showed a significant rise in the proportion of metallic Pt at the surface, in comparison to the reduced catalyst, with fractions close to 70 ± 5% for Pt^0^, suggesting a lack of significant interaction between the Pt and the surface species on the carbon support. The formation of larger particles during the reaction, as indicated by the lower dispersion in the samples after use (Table 1), might lower the influence of nearby species on the electronic properties of the Pt nanoparticles. Additionally, the decreased heteroatom content on the carbon surface may contribute to a reduced interaction between the doped support and the Pt nanoparticles. Furthermore, it has been outlined that such catalytic systems should be preferentially investigated in situ/operando.^72^ In order to gain deeper insights into the local environment of platinum and its oxidation state during the APR reaction, in situ XAS-XRD experiments were conducted.

#### 3.2.3 In situ XAS-XRD Characterization

In situ XAS-XRD measurements at the Pt L_3_ edge were carried out to identify the platinum species in the catalyst formed during reduction and APR. The experimental procedure is outlined in Figure S18. During the experiment, XANES profiles were taken in two stages: first, during the reduction of the catalyst at 300 °C under 5% H_2_/He flow for an hour, and second, during the APR of EG at 225 °C, 30 bar, and WHSV of 9 h^–1^ for 2 h. Additionally, EXAFS and XRD data were collected at the start and end of each stage at 50 °C. Figure S19 shows XANES spectra of the fresh, reduced, and spent catalysts. EXAFS fitting parameters and fitted data analyzed in this section are presented in the Supporting Information Table S6 and Figures S20–S24.

**Figure 5:**
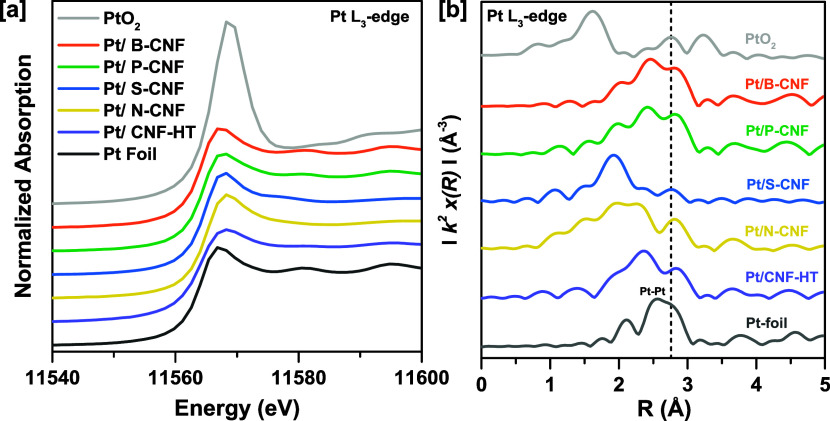
(a) XANES spectra at the Pt L_3_-edge of reduced Pt/X-CNF catalysts in comparison to Pt foil and PtO_2_ references. Spectra are vertically offset to allow for better visualization. (b) *k*^2^-weighed Pt L_3_-edge FT-EXAFS data of reduced Pt/X-CNF in *R* space. The vertical line at 2.76 Å indicates the position of the backscattering peak corresponding to the first Pt–Pt shell of bulk metallic Pt.

The Pt L_3_ XANES spectra of the Pt/X-CNF catalysts presented in Figure 5a indicate that the catalysts are mostly reduced after exposure to H_2_ flow at high temperatures. Observable alterations can be noticed between the intensities and shape of the spectra compared to those of the Pt foil, which can be attributed to the differences in the chemical environment surrounding the absorbing atom. Comparison of the EXAFS data of the Pt foil with the Pt/doped-CNF catalysts indicates the presence of scatterers in the samples, including Pt–Pt and Pt interacting with lighter elements different from oxygen represented in the lower range of the atomic distances, observed by contributions below 2.3 Å.

**Figure 6:**
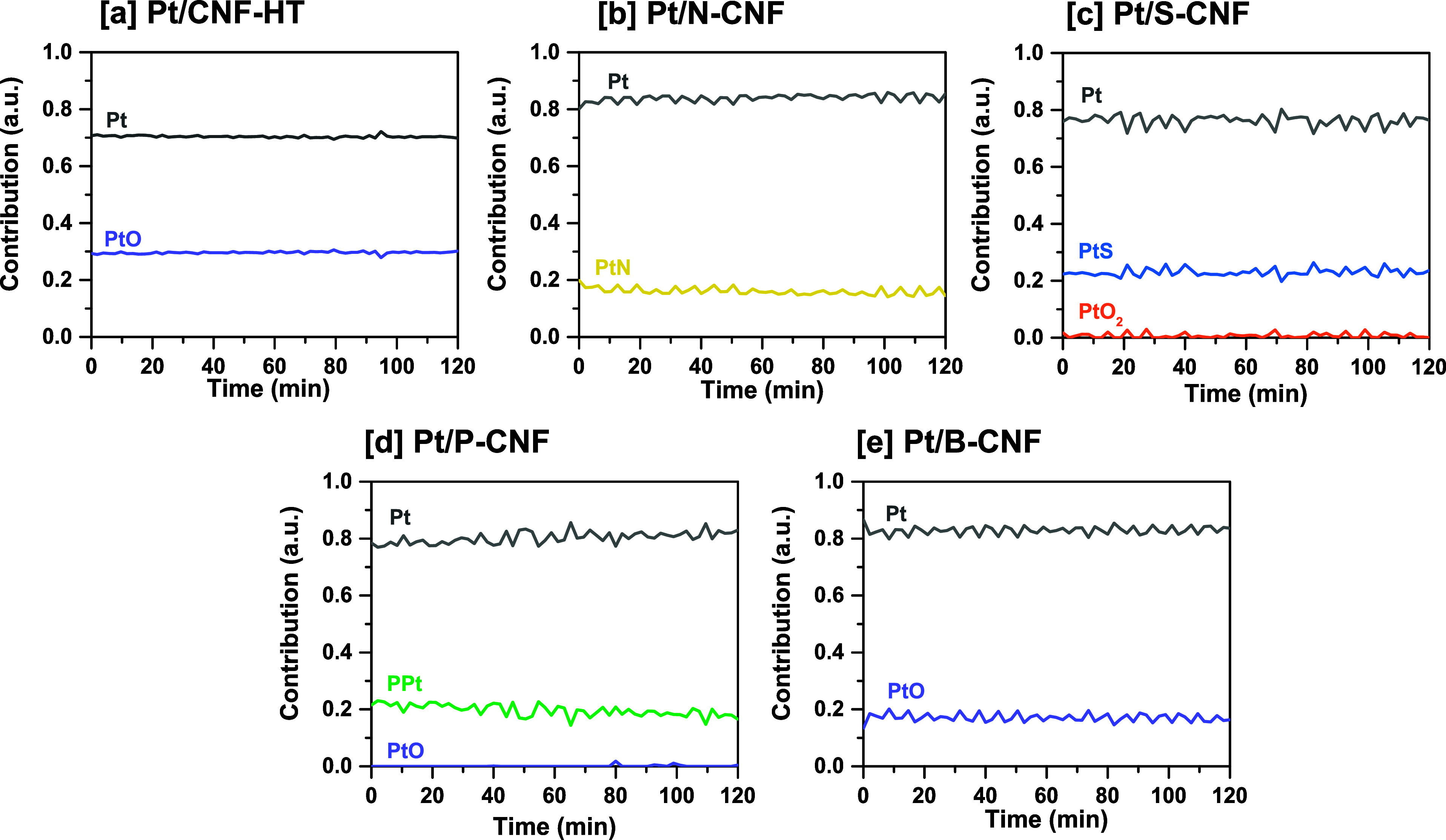
MCR-ALS results from the normalized XANES spectra at the Pt L3-edge acquired during APR of EG, including the contribution of the species according to standards. (a) Pt/CNF-HT, (b) Pt/N-CNF, (c) Pt/S-CNF, (d) Pt/P-CNF, and (e) Pt/B-CNF. The normalized XANES spectra and minimal error of the contribution plot are presented in Figure S25.

From the MCR analysis conducted on the XANES spectra collected during APR (Figure 6), two main platinum species were identified in each catalyst. A significant contribution from Pt^0^ was observed, with a slight increase during APR which is consistent with the XPS results of the spent catalyst (Figure 4). Furthermore, platinum species directly coordinated to the heteroatoms present in N, S, and P containing supports (PtN, PtS, and PtP) were observed. For B-doped support, no contributions from boron-containing species directly coordinated to Pt was found. However, it is important to realize the limitations of EXAFS in distinguishing Pt–C, Pt–N, and Pt–O coordination due to the similar scattering amplitudes of C, N, and O. Therefore, a cautious interpretation of the results is necessary.

The Pt/CNF-HT catalyst exhibited a stable Pt^0^ and Pt^2+^ contribution, with slight sintering of the platinum particles. This was indicated by an increase in the coordination number after APR, from 8.8 to 9.8 in the EXAFS fitting, representing Pt–Pt and Pt–O scattering paths in the catalyst at 2.75 and 1.98 Å bond lengths (Table 2). Small Pt particles are often associated with a contraction of the interatomic distance due to increased electron density on the respective atoms by dehybridization of the spd metal orbital,^73,74^ as observed for this catalyst with 0.03 Å shorter bond length than that for bulk Pt. A decrease in dispersion through chemisorption was also observed, as indicated in Table 1, and the presence of larger particles revealed by the STEM images (Figure 2). Additionally, a coordination number of 0.4 of Pt–O is likely due to the presence of a minor fraction of Pt oxide, in agreement with MCR results.

**Table 2:** Coordination Numbers (CN) and Radial Distances (*R*) Determined by EXAFS Fitting of the Pt L_3_-Edge on Reduced and Spent Catalysts after APR of Ethylene Glycol^a^^,^^c^

catalyst	shell	CN		*R* (Å)	
		reduced	spent	reduced	spent
Pt/CNF-HT	Pt–Pt	8.84	9.87	2.74	2.75
	Pt–O	0.43	0.49	1.75	1.74
	Pt–Pt	2.91	1.45	3.89	3.89
Pt/N-CNF	Pt–Pt	7.78	8.92	2.72	2.75
	Pt–N	1.03	0.85	1.97	1.88
	Pt–Pt	0.60	3.11	3.90	3.90
Pt/S-CNF	Pt–Pt	3.92	5.92	2.72	2.72
	Pt–S	1.29	1.15	2.31	2.30
	Pt–O	2.10	1.69	1.95	1.91
Pt/P-CNF^b^	Pt–Pt	9.33	11.63	2.75	2.76
	Pt–P	0.70	0.48	2.27	2.24
	Pt–Pt	1.51	3.64	3.90	3.88
Pt/B-CNF^b^	Pt–Pt	10.28	10.94	2.75	2.76
	Pt–O	0.07	0.24	1.91	1.86
	Pt–Pt	2.52	2.81	3.90	3.89

a Fitting is performed using the Pt, PtO, PtO_2_, PtN, PtS, and PtP crystallographic structures. Complete set of fitting parameters is presented in Table S6 and fitting curves in Figures S20–S24.

b Spectra fitted in *R*-space.

c Estimated accuracy in coordination number, CN: ±10–20%. Interatomic distance *R*: ±0.02 Å.

The fit of the Pt L_3_-edge EXAFS of the Pt/N-CNF catalyst indicated direct Pt–Pt coordination in the reduced and spent catalyst including Pt–Pt contributions at 2.72 and 2.75 Å, respectively, and a contribution around 1.97 and 1.88 Å reflecting Pt bonded to light elements (N/O/C).^75^ The Pt–Pt bond distance is consistent with that of metallic Pt atoms (2.77 Å), although the value after reduction indicated a contraction of the Pt–Pt distance. This might be attributed to metal–support interactions involving a charge rearrangement in the Pt atoms when the atoms in the support, such as N, have a high electron richness, as suggested by Zhang et al.^74^ Thus, a shorter Pt–Pt distance and a larger structural disorder are observed,^76^ reflected in a larger Debye–Waller type factor of 0.014 Å. At APR conditions, a stable behavior can be observed as there is only a slight increase in the contribution from Pt^0^ along with a minor contribution identified as Pt-N.

Similarly, the Pt/S-CNF catalyst presented a decrease in the Pt–Pt bond length, with an interatomic distance of 2.72 Å, suggesting strong interaction between the metal and the electron-rich sulfur-doped support. Furthermore, STEM (Figure 2) revealed the formation of larger particles, in agreement with an increase in the average Pt–Pt coordination number from 3.9 in the reduced catalyst to 5.9 after the reaction. A Pt–S contribution could be identified during APR, with a Pt–S bond distance of 2.30 Å, which closely resembles that of Pt–S bond distances of Pt supported on S-doped carbon nanotubes (CNTs) previously reported in literature.^77^ Note that this was the only catalyst in which Pt^4+^ species were detected, with a minor contribution (<2%) of PtO_2_.

The Pt/P-CNF spent catalyst had a higher average Pt–Pt coordination number of 11.6 compared to the reduced catalyst, which had a Pt–Pt coordination number of 9.3, in agreement with STEM data, indicating that this catalyst contained large Pt particles distributed over the carbon support. A contribution of Pt^0^ increases during APR, with a minor contribution from P–Pt for the Pt/P-CNF catalyst, as revealed from MCR analysis. The Pt–Pt bond distance of the spent catalyst was similar to that of the Pt foil (2.76 Å). Additionally, it was observed that the Pt–P coordination numbers decreased from 0.7 to 0.4, which is associated with the decrease in P content on the carbon support from 1.78 to 0.84% at, as indicated by XPS.

Attempts to incorporate Pt–B scattering paths in the EXAFS fitting for the Pt/B-CNF were unsuccessful, which is consistent with the MCR results showing only contributions from Pt and PtO. A minor increase in the average Pt–Pt coordination number was observed after APR, as also indicated by a decrease in the Pt dispersion (Table 1) evident also from the STEM images (Figure 2). This is consistent with the XPS results, which show that B is mostly present as an oxidized species limiting the interactions between Pt and B.

The XRD patterns of the reduced and spent catalysts after APR are presented in Figure S26. The diffraction peak near 3.84° in all catalysts is associated with the (002) graphite basal plane of the carbon support. The diffractograms of the catalysts Pt/P-CNF and Pt/B-CNF contain peaks at 6.45, 7.45, 10.55, and 12.38° corresponding to the (111), (200), (220), and (311) planes of fcc platinum.^45^ No significant changes were detected in the Pt XRD peaks, suggesting that the sintering observed in other characterization techniques most likely occurred among the smaller Pt particles, which were not detected by XRD.

### 3.3 Effect of Carbon Doping

#### 3.3.1 Catalyst Synthesis

The final properties of a catalyst are influenced by the selected synthesis method: the distribution of the metal precursor within the carbon support holds significant importance in the impregnation technique. The solvent–carbon interactions, as well as the formal charge of the metal complex can play crucial roles for in the catalyst synthesis.^19,59^ For achieving homogeneously distributed small particles on supports, incipient wetness impregnation using an acetone-based anionic platinum precursor solution proved to be more effective in cases where the surface charges of the supports were closer to zero (CNF-HT and N-CNF) than that in the case of CNF doped with P, B, or S, which have a significant negative or positive surface charge (Figure 3e). It seems that stronger electrostatic repulsion/attraction interactions of the metal ions ([PtCl_6_]^2–^) from the charged CNF surface promote aggregation of Pt particles on the carbon support. These observations align with previous studies by Zhu et al.^78^ evaluating the polyol reduction synthesis method. They noticed that the crucial factor for obtaining the desired metal loading is the electronic attraction caused by the potential difference between the CNF surfaces and the metal colloids during catalyst preparation.

#### 3.3.2 Catalytic Activity

**Figure 7:**
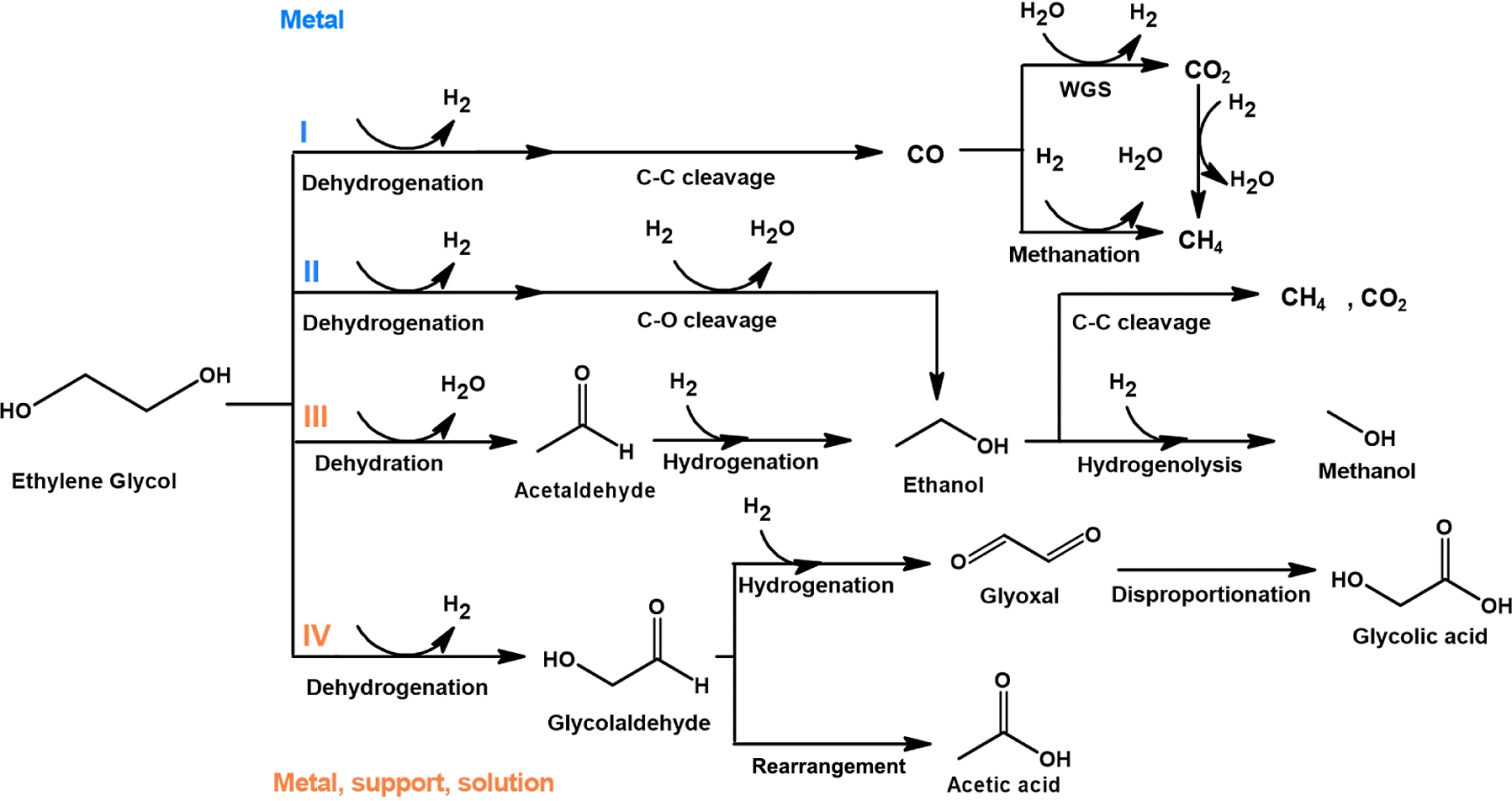
Reaction pathways in APR of EG, based on previous literature.^50,53^

Dumesic et al.^3,7,50,79,80^ extensively discussed the comprehensive reaction pathways of APR of alcohols derived from biomass. Notably, the catalytic activity is significantly influenced by the nature of the support, connecting the conversion of polyalcohols to bifunctional reactions involving both the support and the metal phase.^51^ The pathway leading to the formation of hydrogen and CO_2_ is characterized by a sequence of steps involving EG dehydrogenation, decarbonylation, and subsequent WGS reaction, as shown in Figure 7.^50^ Following these reactions, alkanes are produced through methanation and Fischer–Tropsch synthesis.

After dehydrogenation of EG, the formation of liquid products can occur through C–O bond cleavage that will lead to methanol or the isomerization/rearrangement of the C–O bonds in surface intermediates (glycolaldehyde), potentially resulting in the formation of acetic acid.^80^ Furthermore, glycolaldehyde can undergo additional dehydrogenation, forming glyoxal, which under the influence of an acid-catalyzed Tishchenko reaction, may lead to the formation of glycolic acid through intramolecular disproportionation.^51,53^ Alternatively, EG could undergo dehydration over the metal or acidic catalyst supports to form acetaldehyde, followed by hydrogenation leading to ethanol.^80^ It is likely that ethanol and acetic acid will experience C–C cleavage facilitated by the metal phase, resulting in the formation of methane and CO_2_. Furthermore, methanol may be produced through hydrogenolysis of ethanol or EG.

The high activity of Pt/N-CNF can be attributed to the basic sites available on the catalyst support together with the high platinum dispersion. Gogoi et al.^22^ noted that nitrogen in the carbon structure provided stability to the Ru nanoparticles. It also created a basic environment on the catalyst support that favored the WGS activity during the APR of glycerol. In the case of N-doped carbon supports, the basicity of pyridinic nitrogen groups is relatively high as they exhibit a lone electron pair located in the horizontal sp^2^ hybrid orbital, which is not part of the aromatic system. As a result, it retains its reactivity without being affected by hybridization.^81^ According to studies presented here, pyridinic nitrogen groups are the most prevalent type of nitrogen found on the surface of Pt/N-CNF. This suggests that their presence on the carbon surface can enhance water dissociation, hence resulting in favorable H_2_ production with low CO content, following pathway I (Figure 7).

Previous studies have shown that electronic effects can either hinder or improve the adsorption of reactants on catalysts containing functional groups on carbon supports.^82−85^ According to Wang et al.,^82,83^ during the APR of EG over Pt catalysts supported on carbon nanotubes (CNTs), competitive adsorption occurs between the solvent and the reactant. The high polarity of hydrophilic oxygen-containing supports leads to an increase in the local concentration of water around the support, resulting in a decrease in the hydrogen turnover frequency. Hence, it is expected that oxygen functionalization of Pt-CNF-HT negatively affects the activity during the APR of EG. However, it is anticipated that the heat treatment of the carbon support at 700 °C, following the acid treatment, will decompose acidic groups such as carboxylic acids and carboxylic anhydrides. This will result in a carbon surface containing weak acidic or neutral functional groups such as aldehydes and ethers, as supported by experiments conducted by Chen et al.^86,87^ through TPD studies. This is consistent with the low zeta-potential value (−11 mV) of Pt/CNF-HT at the conditions evaluated (Figure 3). Thus, the presence of oxygen functional groups on the carbon surface facilitates the synthesis of a catalyst with high Pt dispersion, thereby significantly enhancing EG conversion and H_2_ selectivity compared with other catalysts, although it remains less active than Pt/N-CNF.

Negatively charged surface groups of acidic carbons in aqueous solutions, as observed in the zeta potential and NH_3_-TPD measurements for Pt/P-CNF and Pt/B-CNF, are known to facilitate the acid-catalyzed dehydration of EG. This is followed by decarbonylation/decarboxylation and metal-catalyzed hydrogenation reactions that ultimately lead to production of alcohols and alkanes, as shown in Figure 7 pathway III.^7,51^ Furthermore, these surface groups can facilitate the desorption of intermediate species, which can then undergo rearrangement to form organic acids, as observed by Führer et al.,^84^ with enhanced desorption of glycolate in the liquid-phase oxidation of glucose. Hence, acetic acid observed in the liquid product distribution for Pt/P-CNF may be produced as suggested in pathway IV (Figure 7), from the dehydrogenation of EG followed by the rearrangement of intermediates.^7^

Therefore, the low hydrogen yield observed for the Pt/P-CNF (20.1%) and Pt/B-CNF (26.4%) catalysts in comparison to Pt/N-CNF (56.2%) may be attributed to acidic P, B, and O species on the carbon surface, as identified by XPS in the form of oxidized phosphorus species and partially oxidized boron species. These components, particularly the higher oxygen content, could hinder the adsorption of reactants and encourage production of byproducts like alcohols, alkanes, and organic acids. This is reflected in the product distribution shown in Figure 1 with higher yield to hydrocarbons and glycolic acid, following pathways III and IV influenced by the nature of the support.

Another factor to consider is the presence of larger Pt particles, verified through chemisorption of Pt/B-CNF giving 19% Pt dispersion and STEM images of Pt/P-CNF. It seems that larger particle sizes lead to a decrease in the production of H_2_ and CO_2_ (as shown in Table 1) and an increase in the production of light alkanes. This observation agrees with Wawrzetz et al.^51^ Due to platinum sintering, more of the support surface would be available to favor dehydration reactions.

Although the Pt/S-CNF catalyst showed low activity in producing gas-phase products, it was able to generate both ethanol and methanol in the liquid phase. This suggests that the primary catalytic pathway involves cleavage of the C–O bond, followed by hydrogenation, leading to the production of alcohols (pathway II, Figure 7). However, the reduced activity of Pt on S-CNF could be attributed to the presence of metal-sulfur species catalytically inactive for EG reforming, as observed in the in situ XAS characterization, where Pt–S bonds could be identified. Hence, S binding on the Pt sites due to migration on the surface or from leaching into the solution during APR resulted in a lower activity and tuned the selectivity toward alcohols and organic acids.

An increase in the fraction of metallic Pt was observed under APR conditions in the series of catalysts studied, indicating that the platinum particle interaction with the surrounding media causes a reduction of the metal. The generated H_2_ could potentially contribute to the reduction of Pt, and in addition, EG or the produced alcohols in the reaction may convert the Pt^δ+^ species into Pt^0^. Considering that EG is frequently employed to reduce nanoparticle precursors like chloroplatinic acid for Pt nanoparticle formation, a plausible in situ reduction of Pt under hydrothermal conditions is likely.^59^

## 4 Conclusions

The present study showed the strong influence of the properties of the support for particle size and catalytic activity in APR of EG. Specifically, the study unraveled the behavior of platinum nanoparticles in terms of their susceptibility to changes induced by the surrounding chemical environment and metal–support interactions, which affect their electronic properties and ultimately their catalytic activity. Therefore, by understanding the consequences of these modifications, it will be possible to refine the properties of the catalysts, leading to the development of an efficient catalyst for the APR of polyols derived from biomass.

In situ XAS allowed for the identification of Pt heteroatom species and the Pt oxidation state during APR, correlating the Pt local coordination and electronic structure to the catalyst activity and selectivity during the reaction. The findings support the assumption that catalyst activity and selectivity are significantly influenced by the nature of the support and the active phase in the catalysts. Sintering of the platinum nanoparticles was confirmed by STEM images and CO chemisorption, which was further supported by in situ EXAFS results.

Based on the synthesis method and reaction conditions used, Pt/N-CNF appears to be the most favorable catalyst for APR of EG among the catalyst studied. The involvement of strong metal anchoring sites favored the formation of small nanoparticles and limited their growth even under the harsh hydrothermal conditions of APR. Furthermore, the introduction of basic sites on the carbon surface facilitated the formation of H_2_ through the WGS reaction. Hence, a higher catalytic activity and H_2_ selectivity was obtained compared to the Pt-based catalysts supported on carbon nanofibers doped with oxygen, phosphorus, boron, and sulfur. On the other hand, Pt/S-CNF displayed the lowest catalytic activity due to the formation of Pt-S species.

## Supporting Information Available

GAHDof CNFs; band assignments and fitting parameters for the XPS analysis; in situ XAS-XRD APR experiments at SNBL BM31; high-pressure cell used for the APR experiments at the CAT-ACT beamline at the KIT Light Source; parameters and equations applied to evaluate the APR catalyst performance; blank and support experiments; N_2_ physisorption isotherms of CNF subjected to heat treatment and N, S, P, and B doping; BET specific surface area and total pore volume for CNF supports; Raman spectra showing the D and G bands of the CNF subjected to heat treatment and N, S, P, and B doping; Raman intensity ratio between the D and G bands; Pt particle size distributions from STEM images of reduced and spent catalysts, and Pt/B-CNF; EDS mapping for reduced and spent catalysts containing HAADF-STEM images and Pt, C, and O signals; high-resolution XPS of reduced and spent catalysts; surface concentration of individual heteroatom species determined by deconvolution of high-resolution XPS spectra; NH_3_-TPD profiles of the catalysts; fractions of Pt species of reduced and spent catalyst as determined by deconvolution of the Pt 4f spectra in XPS; high-resolution XPS Pt_4f_ spectra; experimental procedure followed at SNBL BM31; Pt L_3_-edge XANES spectra for fresh, reduced, and after APR of EG; normalized XANES spectra at the Pt L_3_-edge acquired during APR of EG and minimal error contribution plot from the MCR-ALS analysis; and XRD patterns recorded during in situ APR of EG (PDF)
