# Supplementary material for: Aqueous Phase Reforming over Platinum Catalysts on Doped Carbon Supports: Exploring Platinum–Heteroatom Interactions
Source: ACS Catal. 2024 Mar 4;14(6):4139–54. doi: 10.1021/acscatal.3c05385 (PMC10949196; doi:10.1021/acscatal.3c05385)
Supplement: Supplementary file 1 — cs3c05385_si_001.pdf [file cs3c05385_si_001.pdf]

Aqueous phase reforming over platinum catalysts on doped carbon supports: Exploring platinum-heteroatom interactions

Supplementary Information

Monica Pazos Urrea[1], Simon Meilinger[1], Felix Herold[1], Jithin Gopakumar[1], Enrico Tusini[2], Andrea De Giacinto[2] , Anna Zimina[3], Jan-Dierk Grunwaldt[2,3], De Chen[1], Magnus Rønning[1]*

[1]Department of Chemical Engineering, Norwegian University of Science and Technology, 7491Trondheim, Norway

[2]Institute for Chemical Technology and Polymer Chemistry, Karlsruhe Institute of Technology, Engesserstraße 20, 76131 Karlsruhe, Germany

[3] Institute of Catalysis Research and Technology, Karlsruhe Institute of Technology, Hermann-von-Helmholtz Platz 1, 76344 Eggenstein-Leopoldshafen, Germany

*Corresponding Author: Magnus Rønning, Email: magnus.ronning@ntnu.no

1. EXPERIMENTAL

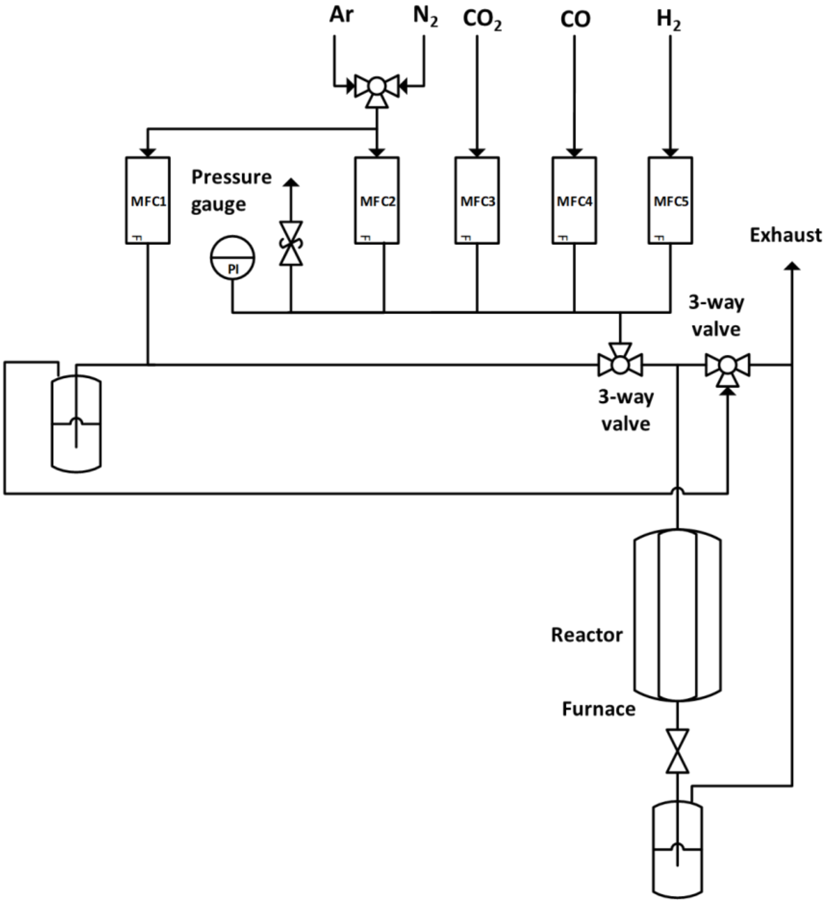

Figure S 1. Schematic diagram of the setup used for gasification-assisted heteroatom doping (GAHD) of carbon nanofibers (CNF).

XPS

The N 1s deconvolution was constrained with a set value of the full width at half maximum (FWHM) to 1.5 ± 0.1 eV, and the shape factors were maintained at approximately 0.5, equal for all functions during the fitting process. A comprehensive list of the species present in the spectra and their corresponding binding energies can be found in Table S1.

The S 2p contribution were deconvoluted considering $2p_{1/2}$ and $2_{3/2}$ S species with doubles of two pseudo-Voigt profiles with a fixed intensity ratio of 0.55±0.5 and a set relative binding energy of 1.18 eV. The FWHM was limited within the range of 1.2±0.2 eV. The shape factors were consistently kept at around 0.5 during the fitting process across all tasks.

The P 2p contribution included $2p_{1/2}$ and $3P_{3/2}$, represented by two pseudo-Voigt profiles with a fixed intensity ratio of 0.65 ± 0.5 and set relative binding energy of 0.84 eV. The FWHM was constrained to 1.5f ± 0.1 eV. The shape factors were maintained equal for all functions during the fit and were typically close to a value of 0.5.

The B 1s spectrum consisted of four components. The FWHM was constrained to 0.75 ± 0.5 eV. The shape factors were kept equal for all functions during the fit and were typically close to a value of 0.5.

For the analysis of Pt species, the distance between the spin-orbit splitting is set to 3.3 eV to represent $4f_{7/2}$ and $4f_{5/2}$, and the intensity ratio was constrained to 0.8 ± 0.05. FWHM and the shape factor of Pt 4f were set to be equal for all the functions during the fitting.

Table S 1. Band assignments and fitting parameters for the XPS analysis

**Contribution**	**Species**	**Binding energy (eV)**	**Reference**
N 1s	Oxidized N	403.4 ± 0.2	[1,2]
	Quaternary N	401.0 ± 0.2	
	Pyrrolic N	399.7 ± 0.2	
	Pyridinic N	398.3 ± 0.2	
S 2p	Aliphatic sulfides	162 ± 0.3	[3,4]
	Aromatic sulfides (Thiophenes)	164.0 ± 0.2	
	Oxidized sulfur species (sulfoxides)	167.5 ± 0.5	
P 2p	Reduced phosphorus (C_3PO_1)[a]	132.6 ± 0.2	[3,5,6]
	Oxidized phosphorus ($C_{0-1}PO_{3-4}$)	134.0 ± 0.2	
B 1s	BC_3	190.0± 0.2	[7,8]
	BC_2O	190.7± 0.2	
	BCO_2	191.9± 0.2	
	B_2O_3	193.2± 0.2	
Pt 4f	Pt $4f_{7/2}$ - Pt^o	71.7 ± 0.2	[9]
	Pt $4f_{7/2}$ - Pt^{+2}	72.6 ± 0.2	
	Pt $4f_{7/2}$ - Pt^{+4}	74.4 ± 0.2	

[a]Triphenylphosphin oxide as model compound [6]

In situ XAS-XRD setup for APR

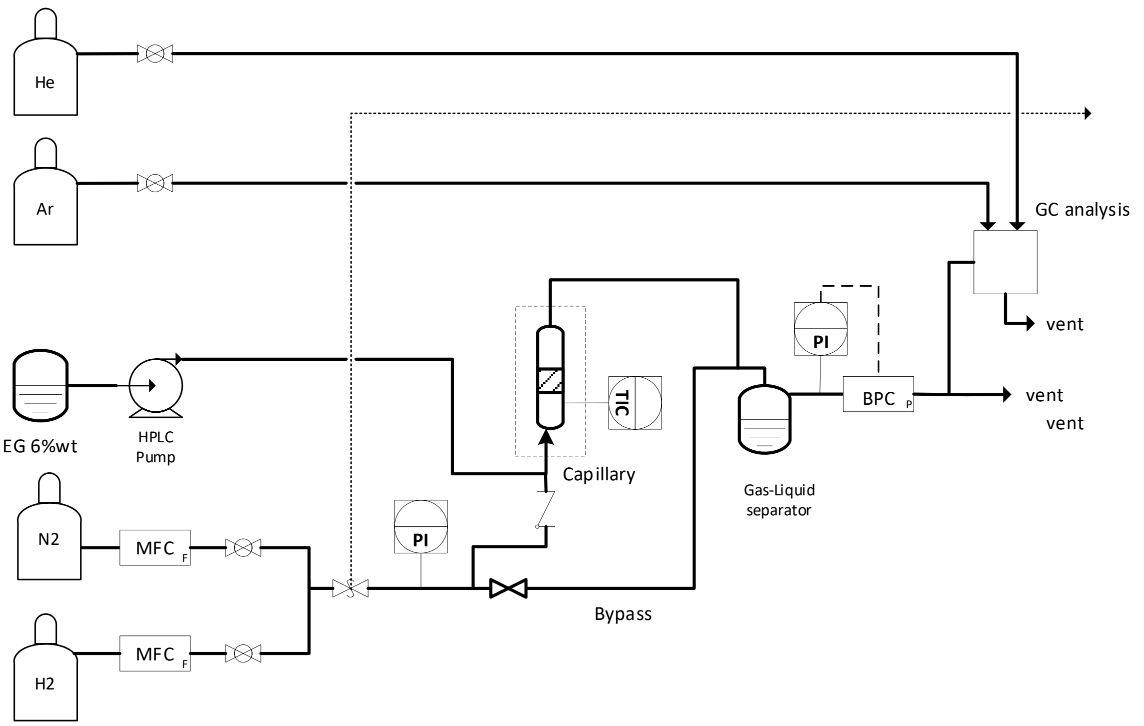

Figure S 2. Scheme of the setup used for the *in-situ* XAS-XRD APR experiments at SNBL BM31.

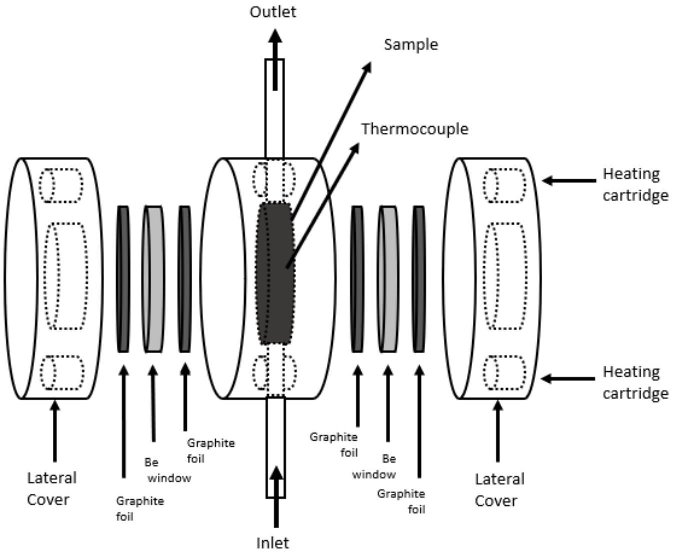

Figure S 3. Scheme of the high pressure cell used for the APR experiments at the CAT-ACT beamline at the KIT Light Source, based on reaction cell in [9,10]

Table S 2. Parameters and equations applied to evaluate APR catalyst performance.

Equation	Parameter	Formula
1	Conversion	$X_{EG}(\%) = \frac{mol_{EG\ initial} - mol_{EG\ final}}{mol_{EG\ initial}}$
2	Hydrogen selectivity – Carbon based	$S_{H2}(\%) = \frac{mol\ H_{2\ produced}}{C_{total,g}{}^{a}}\ x\frac{1}{R^{b}}x100$
3	Carbon based selectivity	$S_{i,g}\ (\%) = \frac{C_{i,g}{}^{c}}{C_{total,g}{}^{a}}\ x\ 100$
4	Hydrogen site time yield	$STY_{H2}(min^{-1}) = \frac{H_{2\ production\ rate}{}^{d}\ Mw_{Pt}{}^{e}}{D_{Pt}{}^{f}W_{cat}{}^{g}X_{Pt}{}^{h}}$
5	Hydrogen Production	$Production_{H_2}\ (\frac{mol}{g_{pt}}) = \frac{mol\ H_{2\ produced}}{W_{cat}{}^{g}X_{Pt}{}^{h}}$
6	Hydrogen yield	$Y_{H2}(\%) = \frac{mol\ H_{2\ produced}}{mol_{EG\ initial}}\ x\frac{1}{R^{b}}x100$
7	Carbon yield to liquid products	$C_{i,L}\ (\%) = \frac{C_{i,L}{}^{j}}{C_{EG\ inital}{}^{k}}\ x\ 100$
8	Carbon yield to gaseous products	$C_{i,g}\ (\%) = \frac{C_{i,g}{}^{c}}{C_{EG\ inital}{}^{k}}\ x\ 100$
9	Carbon yield to hydrocarbons	$Y_{HC}\ (\%) = \frac{\sum C_{c1-c6,g}{}^{h}}{C_{EG\ inital}{}^{k}}\ x\ 100$

[a] Total carbon present in the gas phase
[b] Stoichiometric reforming ratio for EG $R = H_2/CO_2 = 5/2$
[c] Total carbon in each gaseous product (i)
[d] Moles of H_2 produced divided by the duration of the experiment (120 min)
[e] Molecular weight of Pt
[f] Pt dispersion estimated by CO chemisorption (%)
[g] Amount of catalyst (g)
[h] Weight fraction of Pt in the catalyst (wt%)
[j] Total carbon in each liquid product (i)
[k] Total carbon contained in the feed stream
[h] Besides alkanes (C1 – C6), ethene was also occasionally detected and included.

2. RESULTS AND DISCUSSION

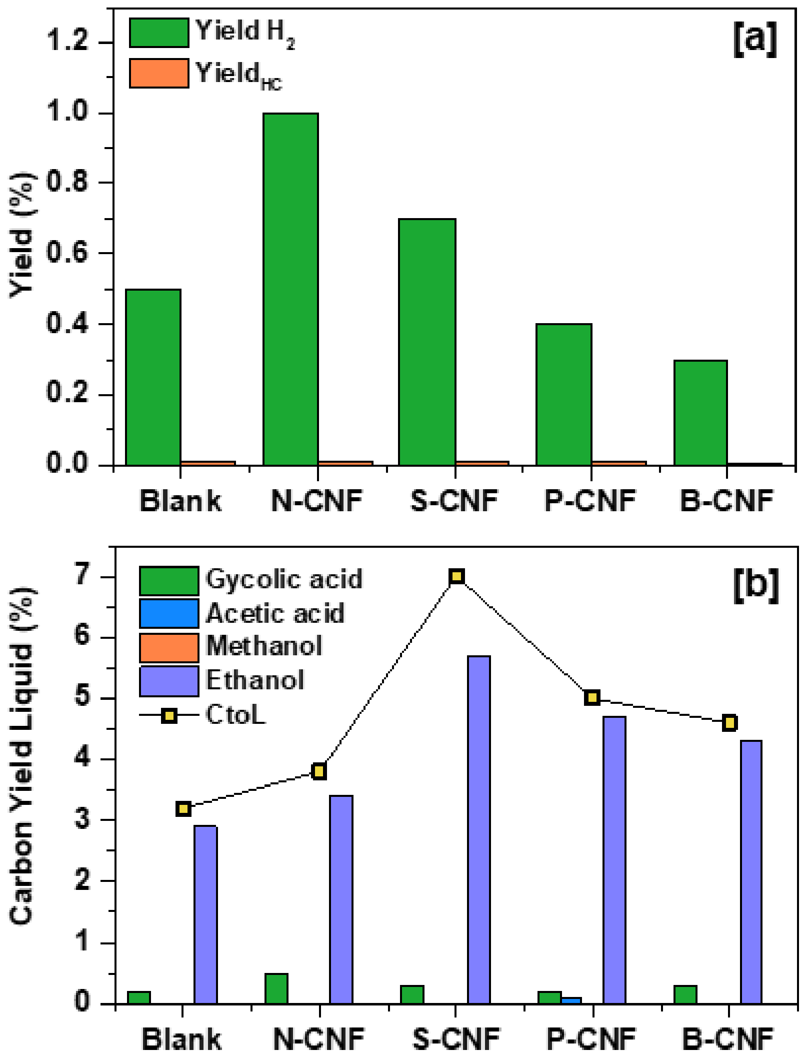

Figure S 4. Blank and support experiments of APR at 250 °C for 2 h [a] Hydrogen and hydrocarbon yields (%) . [b] Carbon yield of liquid products (%)

Table S 3. APR of ethylene glycol (6 %wt) at 250 °C and 26 bar initial pressure for 2h at batch conditions. The carbon balance was confirmed to a degree of 88-100 % for all measurements.

Catalyst	Conversion (±1.5%)	STY_{H2} (min^{-1})		Hydrogen production (± 1.2 $molH_2/g_{Pt}$)	Carbon Yield to liquid (±0.8%)	Carbon balance (%)	S_{H2} (±2%)	SC_{2+} (±0.2%)	Gas phase composition (mol %)			
		Before[a]	After[b]						H_2	CO	CO_2	C_{2+}
CNF-HT	0	-	-	-	3.2	97	-	60	98.4	0.0	0.0	1.6
N-CNF	0	-	-	-	3.8	96	-	27	99.2	0.0	0.0	0.6
S-CNF	0	-	-	-	7.0	93	-	50	98.7	0.0	0.0	1.3
P-CNF	0	-	-	-	5.0	95	-	50	97.5	0.0	0.0	3.5
B-CNF	-1	-	-	-	4.6	94	-	48	98.7	0.0	0.0	1.3
Pt/CNF-HT	21	31±1	52±2	8.9	7.9	88	94	4	70.2	0.3	28.5	1.0
Pt/ N-CNF	26	45±1	63±2	11.3	6.9	90	96	2	70.7	0.2	28.5	0.6
Pt/S-CNF	3	6±0	14±1	1.0	11.8	88	66	23	64.1	2.9	26.9	6.0
Pt/P-CNF	15	-	-	3.4	11.6	91	78	12	66.6	0.3	29.8	3.3
Pt/B-CNF	14	35±1	73±1	4.1	7.6	92	90	8	69.4	0.4	28.2	2.1

[a] Based on the CO uptake before APR of ethylene glycol (6 %wt) at 250 °C and 26 bar initial pressure for 2h
[b] Based on the CO uptake after APR of ethylene glycol (6 %wt) at 250 °C and 26 bar initial pressure for 2h

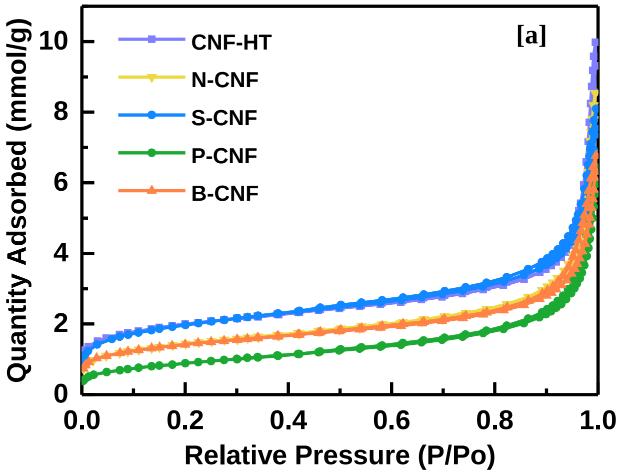

[b]

Support	Surface area (m^2/g)	Total pore volume (ml/g)*
CNF-HT	159	0.13
N-CNF	114	0.11
S-CNF	156	0.13
P-CNF	71	0.08
B-CNF	113	0.10

*The interstitial space between adjacent fibers determines the pores in this type of nanostructured material.

Figure S 5. [a] N_2 physisorption isotherms of CNF subjected to heat treatment, N, S, P and B doping. [b] BET specific surface area and total pore volume for all CNF supports estimated by single-point desorption at p/p0 = 0.9

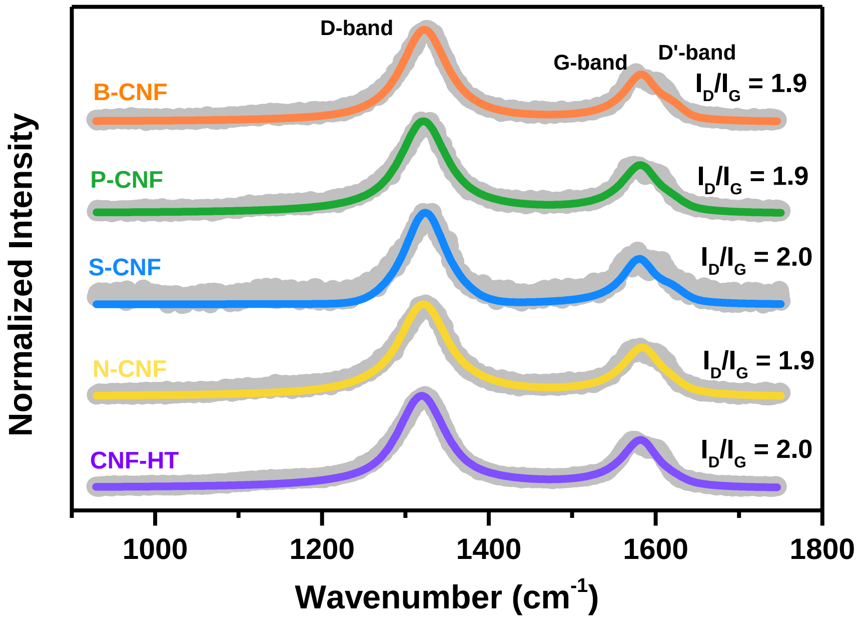

Figure S 6. Raman spectra showing the D and G bands of the CNF subjected to heat treatment, N, S, P and B doping.

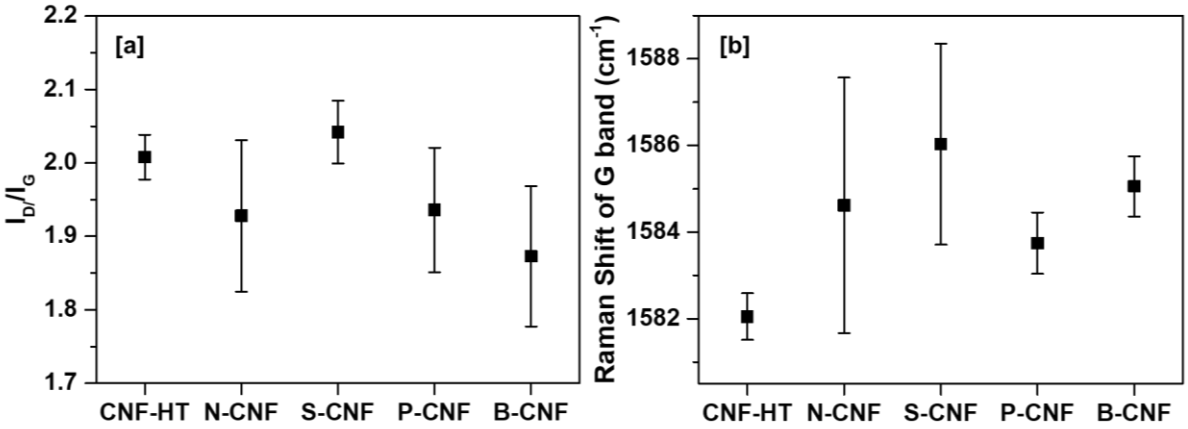

Figure S 7. [a] Raman intensity ratio between the D and G bands [c] Raman shift of the G band of CNF samples subjected to heat treatment, N, S, P and B doping.

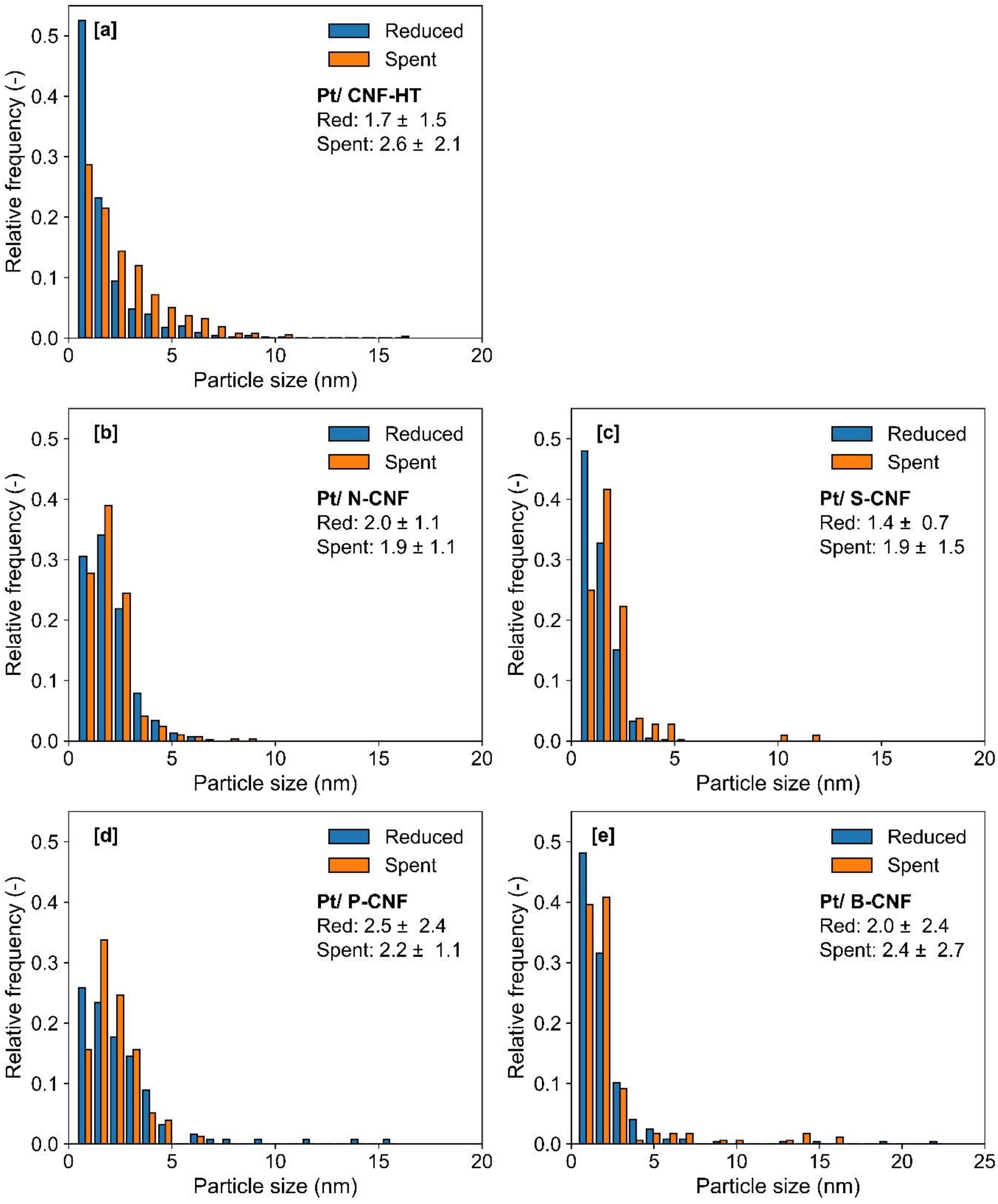

Figure S 8. Pt particle size distributions from STEM images of reduced and spent [a] Pt/CNF–HT, [b] Pt/N-CNF–N, [c] Pt/S-CNF, [d] Pt/P-CNF, [e] Pt/B-CNF.

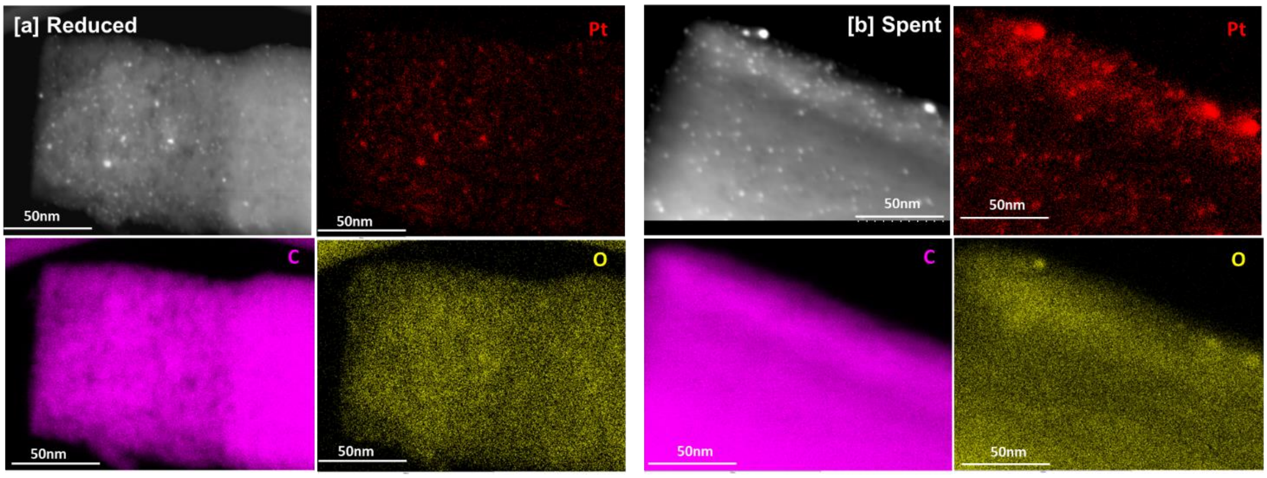

Figure S 9. EDS mapping for [a] reduced and [b] spent Pt/CNF-HT catalyst containing HAADF-STEM images and Pt, C, and O signals.

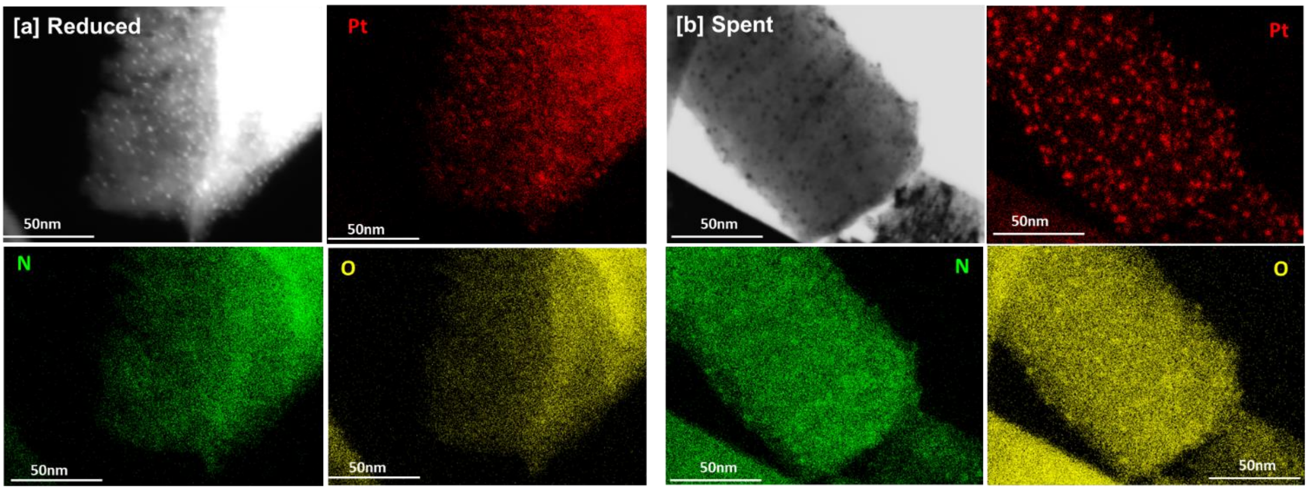

Figure S 10. EDS mapping for [a] reduced and [b] spent Pt/N-CNF catalyst containing HAADF-STEM images and Pt, N, and O signals.

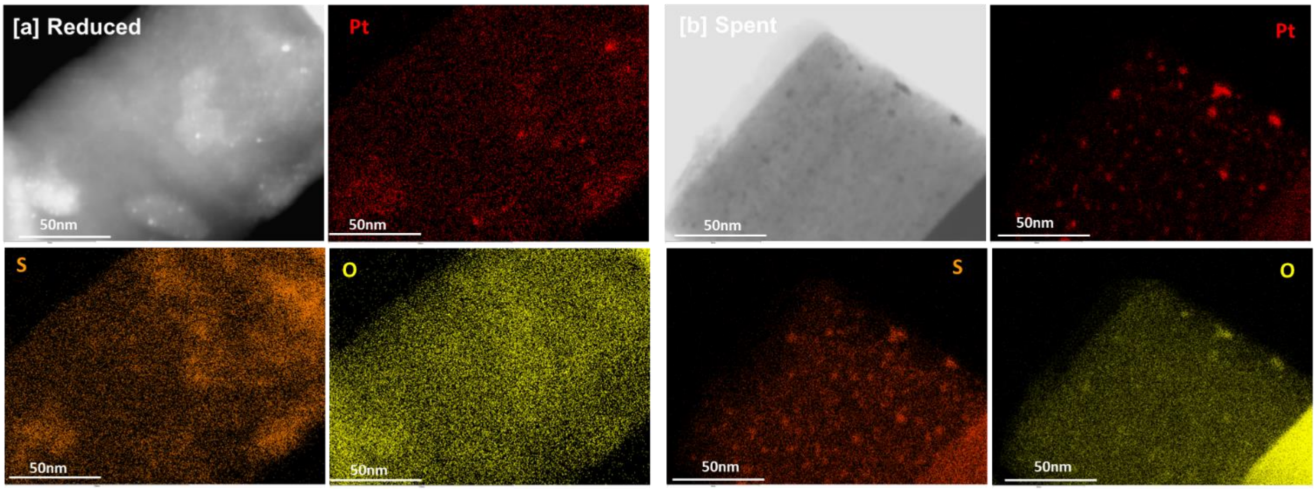

Figure S 11. EDS mapping for [a] reduced and [b] spent Pt/S-CNF catalyst containing HAADF-STEM images and Pt, S, and O signals.

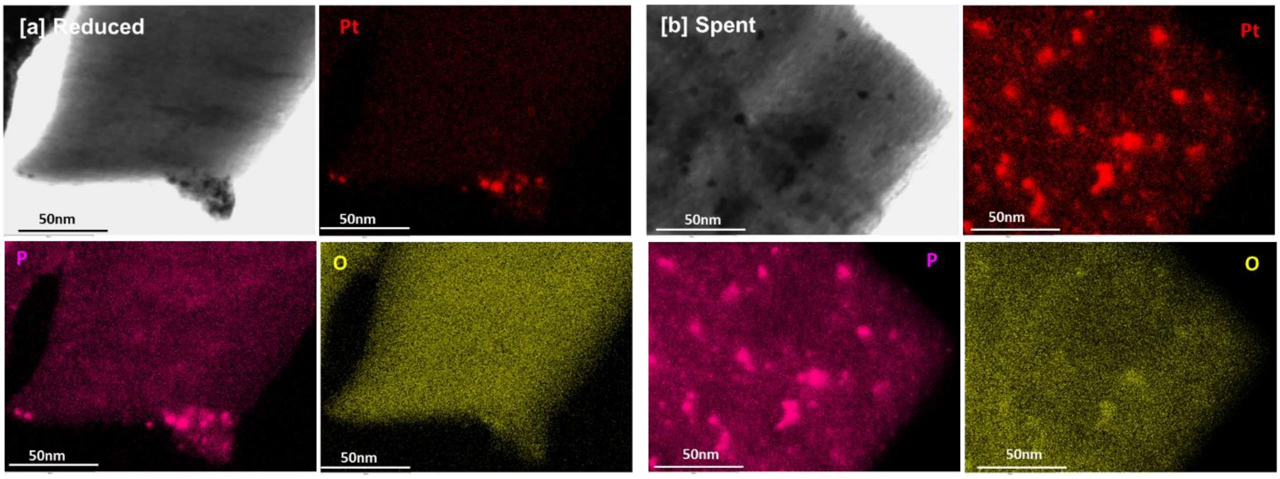

Figure S 12. EDS mapping for [a] reduced and [b] spent Pt/P-CNF catalyst containing HAADF-STEM images and Pt, P, and O signals.

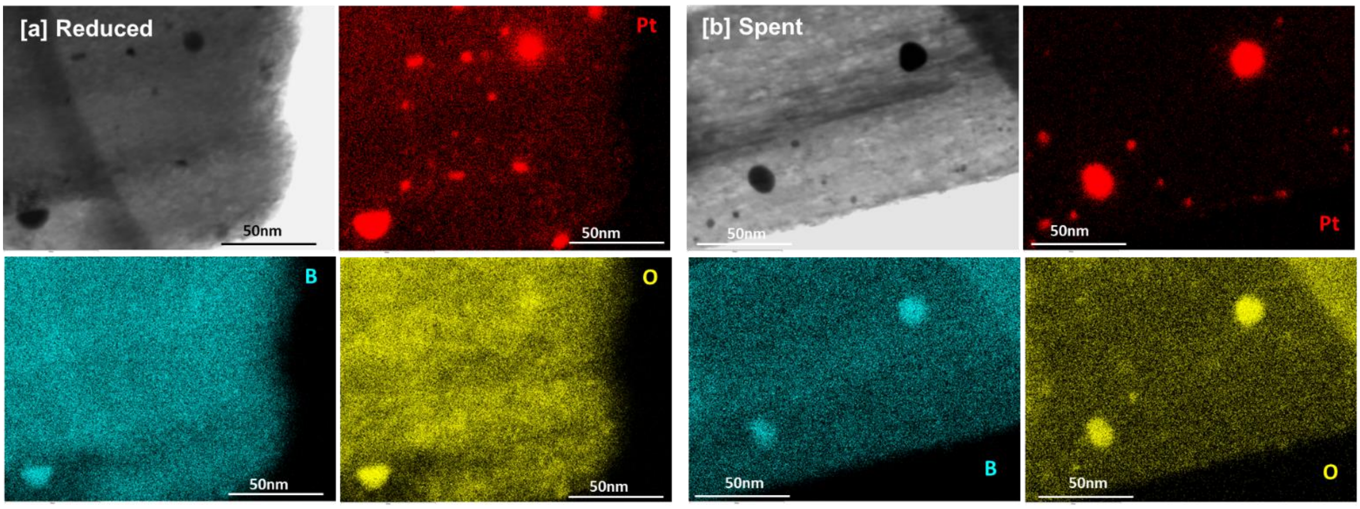

Figure S 13. EDS mapping for [a] reduced and [b] spent Pt/B-CNF catalyst containing HAADF-STEM images and Pt, B, and O signals.

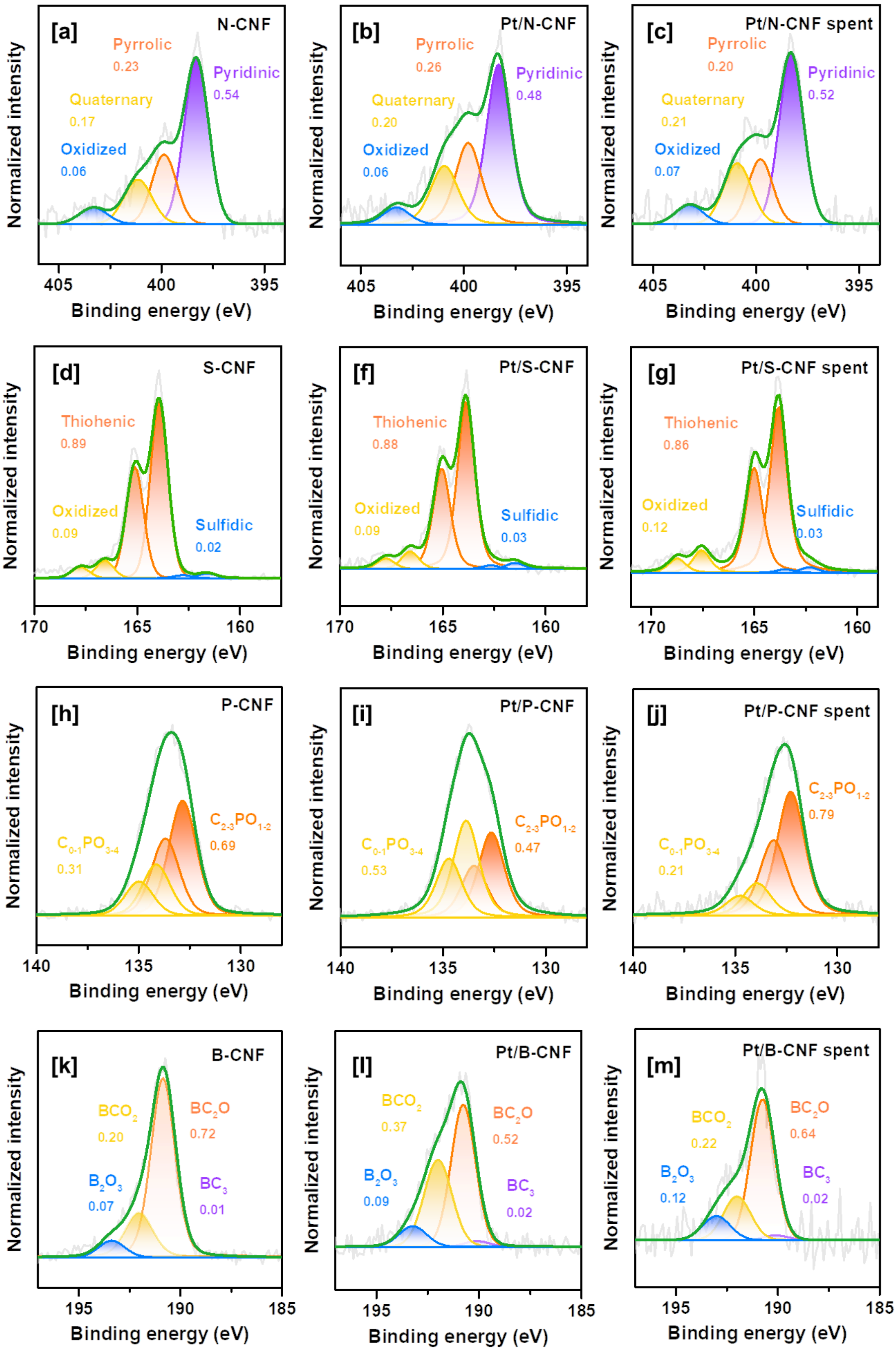

Figure S 14. High-resolution XPS [a, b, c] N 1s of N-CNF, Pt/ N-CNF reduced and Pt/ N-CNF spent [d, f, g] S 2p of S-CNF, Pt/ S-CNF reduced and Pt/ S-CNF spent [h, i, j] P 2p of P-CNF, Pt/ P-CNF reduced and Pt/ P-CNF spent [k, l, m] B 1s of B-CNF, Pt/ B-CNF reduced and Pt/ B-CNF spent.

Table S 4. Surface concentration of individual heteroatom species determined by deconvolution of high-resolution XPS spectra.

Sample	O* [at-%]	Heteroatom* [at-%]	Heteroatom species (%)			
			**C=O**	**C-O**	**H_2O ads**	
CNF-HT	2.8	-	0.40	0.57	0.03	
Pt/ CNF-HT	3.1	-	0.42	0.51	0.07	
Pt/CNF-HT spent	3.9	-	0.37	0.59	0.04	
			**Pyridinic N**	**Pyrrolic N**	**Quaternary N**	**Oxidized N**
N-CNF	2.2	1.8	0.54	0.23	0.17	0.06
Pt/ N-CNF	1.8	1.6	0.48	0.26	0.20	0.06
Pt/ N-CNF spent	1.7	1.9	0.52	0.20	0.21	0.07
			**Sulfides**	**Thiophenic S**	**Oxidized S**	
S-CNF	0.9	1.8	0.02	0.89	0.09	
Pt/S-CNF	3.1	1.6	0.03	0.88	0.09	
Pt/S-CNF spent	3.0	1.3	0.03	0.86	0.12	
			**Reduced P**	**Oxidized P**		
P-CNF	5.5	2.1	0.69	0.31		
Pt/P-CNF	4.7	1.9	0.47	0.53		
Pt/P-CNF spent	3.1	0.8	0.79	0.21		
			**BC_3**	**BC_2O**	**BCO_2**	**B_2O_3**
B-CNF	7.9	3.2	0.01	0.72	0.20	0.07
Pt/B-CNF	6.9	2.0	0.02	0.52	0.37	0.09
Pt/B-CNF spent	4.5	0.8	0.02	0.64	0.22	0.12

*Surface concentrations without considering Pt.

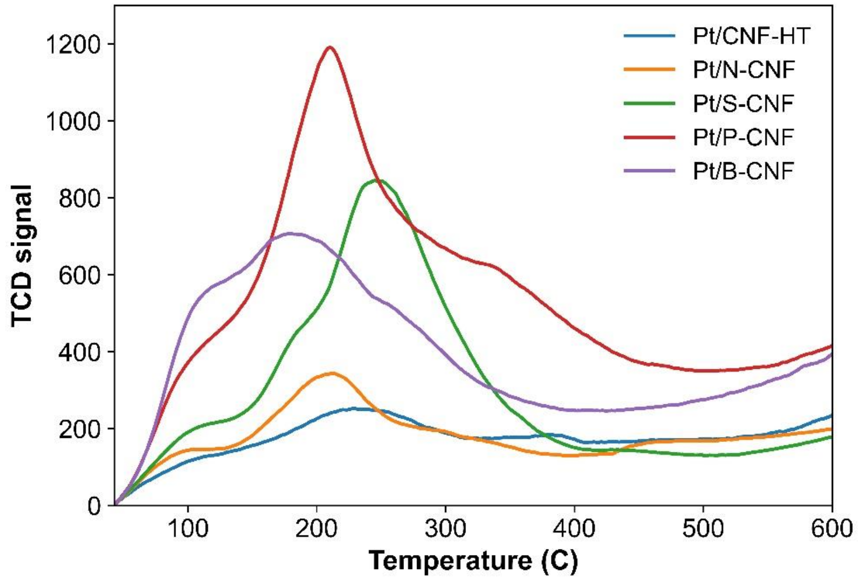

Figure S 15. NH_3 temperature-programmed desorption profiles of Pt/CNF-HT, Pt/N-CNF, Pt/S-CNF, Pt/P-CNF and Pt/B-CNF.

Table S 5. Fractions of Pt species of reduced and spent catalyst as determined by deconvolution of the Pt 4f spectra in XPS.

Catalyst	Pt $4f_{7/2}$ binding energy (eV)	Ratio $Pt^0/(Pt^{2+/4+})$	Platinum species fractions		
			Pt^0	Pt^{2+}	Pt^{4+}
Pt/CNF-HT	72.0	1.1	0.53	0.32	0.15
Pt/N-CNF	72.0	0.6	0.37	0.51	0.12
Pt/S-CNF	72.0	0.8	0.44	0.44	0.12
Pt/P-CNF	71.7	1.3	0.56	0.34	0.10
Pt/B-CNF	71.6	1.9	0.66	0.24	0.10
Pt/CNF-HT spent*	71.8	1.8	0.64	0.24	0.12
Pt/N-CNF spent*	71.7	1.9	0.65	0.26	0.09
Pt/S-CNF spent*	71.9	2.7	0.73	0.18	0.09
Pt/P-CNF spent*	71.9	3.0	0.75	0.17	0.08
Pt/B-CNF spent*	71.6	3.2	0.76	0.18	0.06

*Spent - After 2hours of aqueous phase reforming of EG at 250°C and 26 bar of initial pressure.

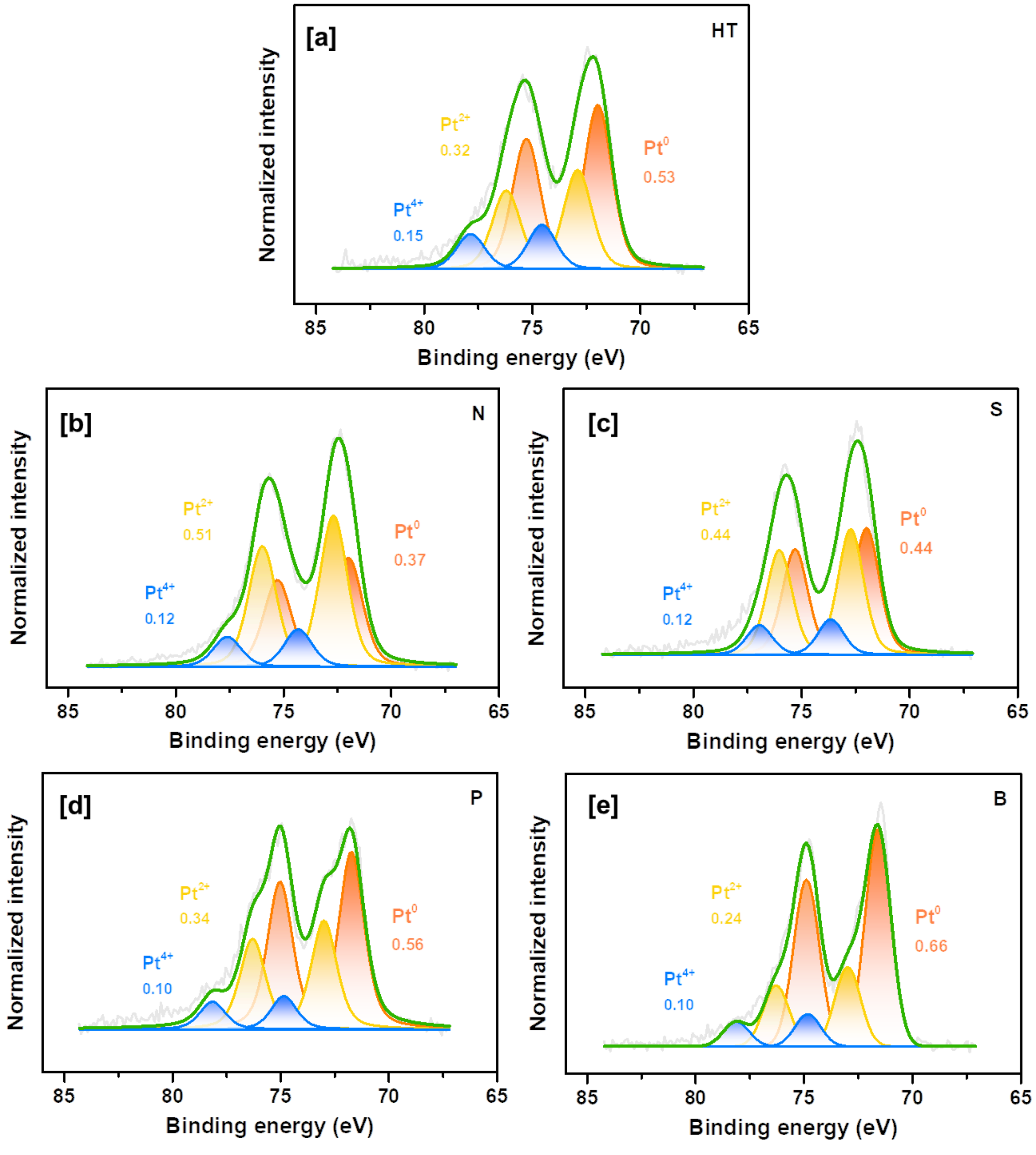

Figure S 16. High-resolution XPS Pt_{4f} spectra of [a] Pt/ CNF-HT [b] Pt/ N-CNF [c] Pt/S-CNF [d] Pt/P-CNF and [e] Pt/B-CNF reduced catalysts.

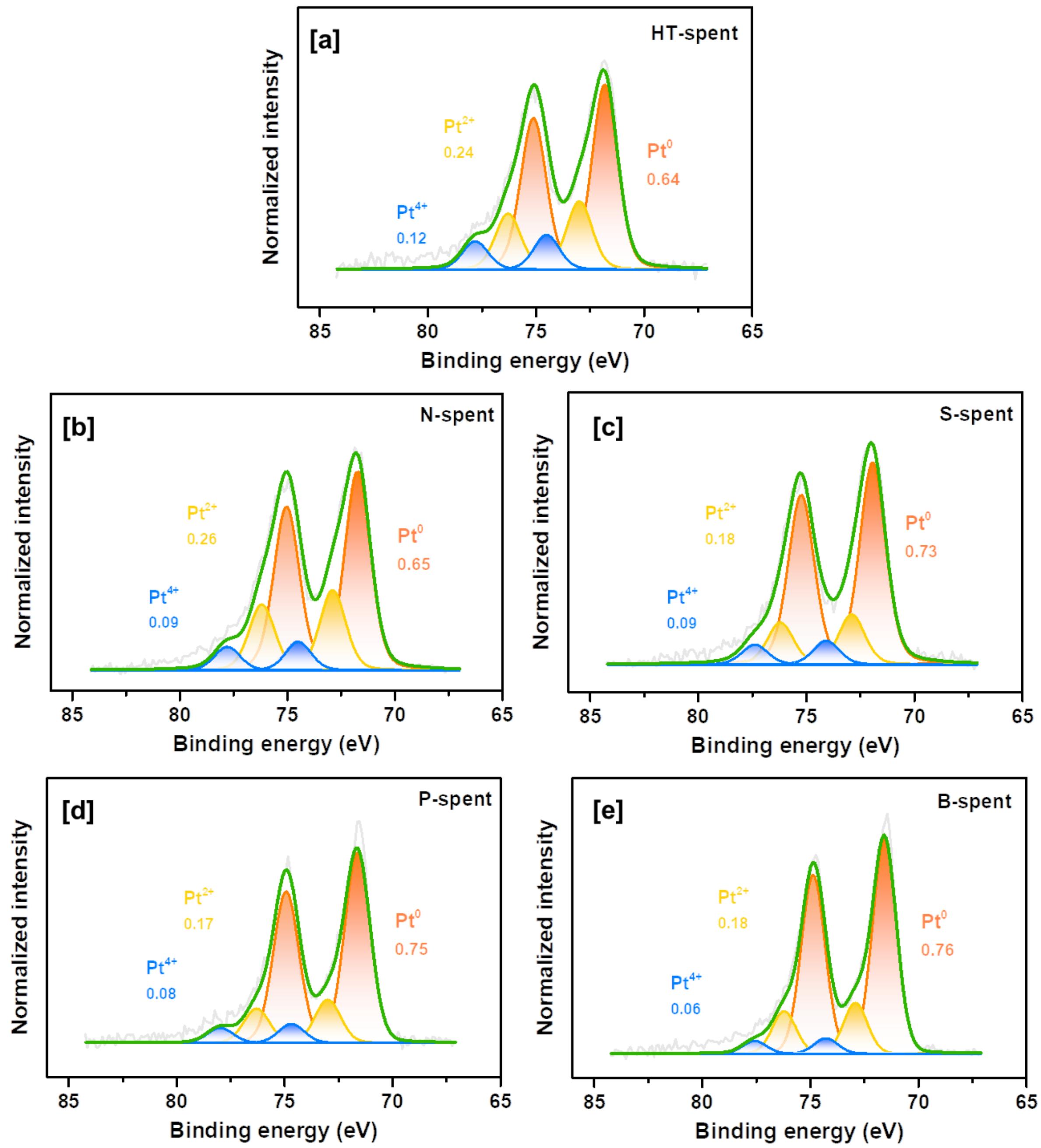

Figure S 17. High-resolution XPS Pt_{4f} spectra of [a] Pt/ CNF-HT [b] Pt/ N-CNF [c] Pt/S-CNF [d] Pt/P-CNF and [e] Pt/B-CNF spent catalysts.

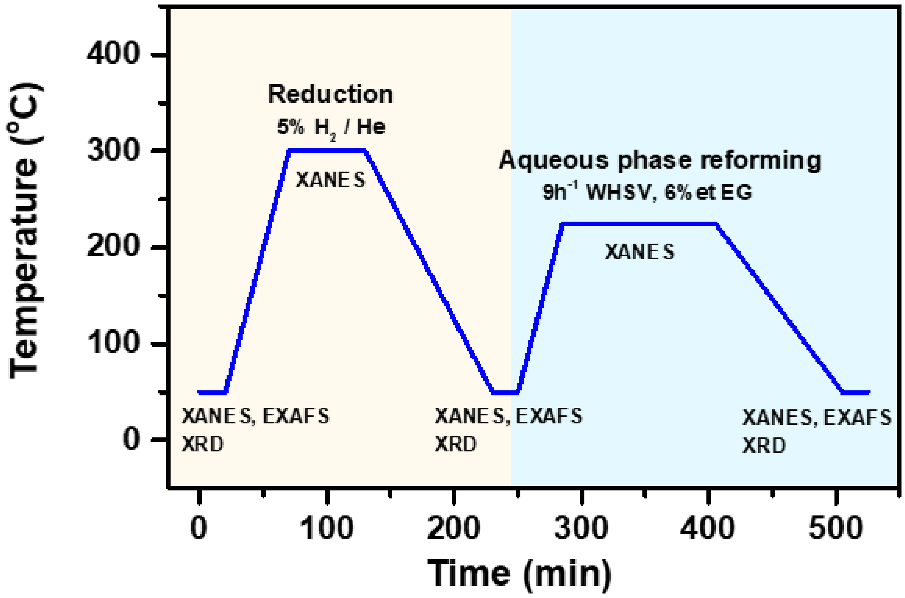

Figure S 18. Experimental procedure followed at SNBL BM31. Step 1 (yellow region) Catalyst reduction at 300°C in 5% H_2 flow for 1 hour. Step 2. (blue region) Aqueous phase reforming of 6%wt ethylene glycol solution at 225°C and 30 bar for 2 hours. (WHSV = $9h^{-1}$)

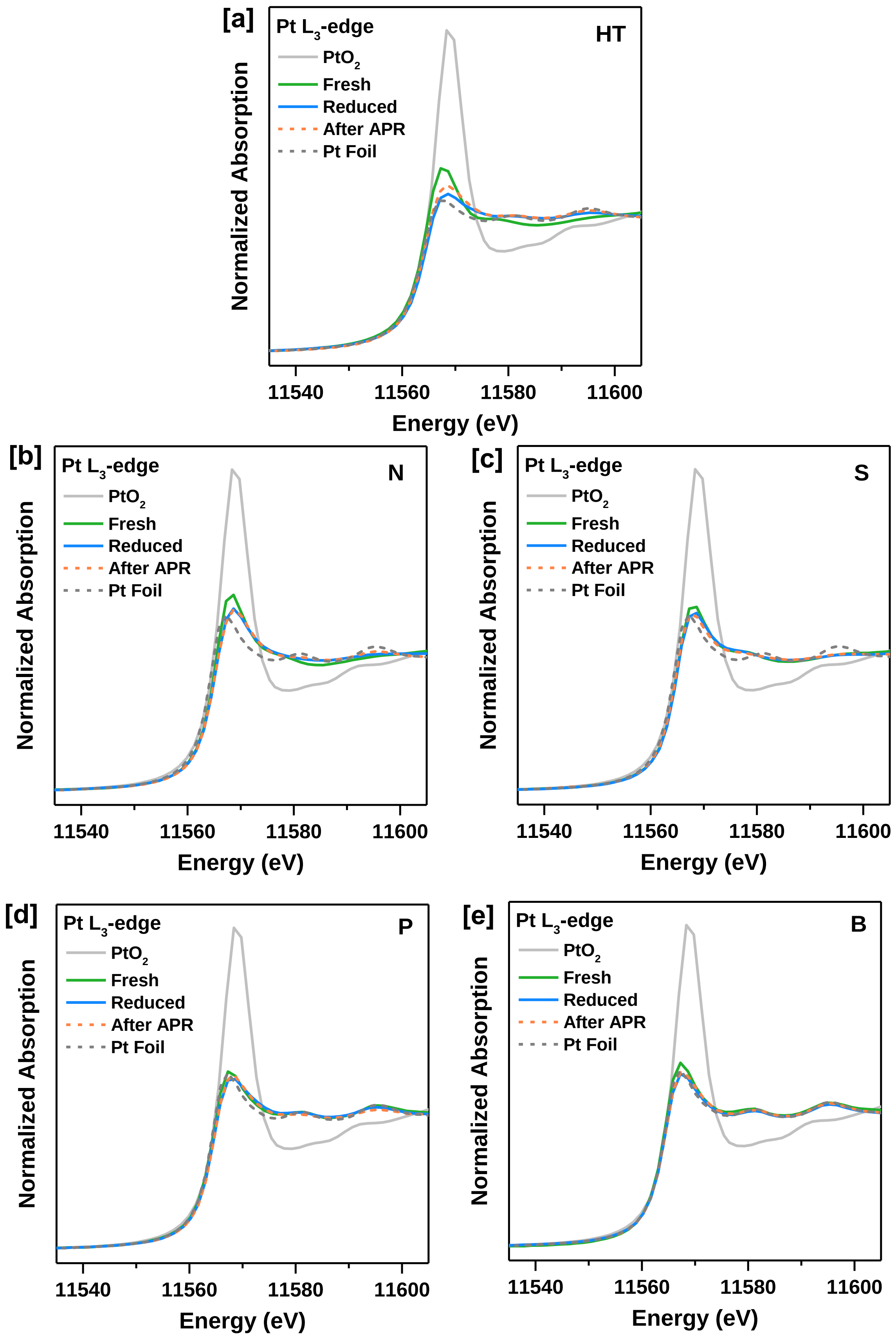

Figure S 19. Pt L_3-edge XANES spectra for fresh, reduced and after APR of ethylene glycol at 225°C and 30 bars (WHSV = $9h^{-1}$) catalysts. [a] Pt/CNF-HT [b] Pt/N-CNF [c] Pt/S-CNF [d] Pt/P-CNF [e] Pt/B-CNF

Table S 6. Coordination numbers (CN), radial distances (R), Debye–Waller type factor (σ^2), and R-factor determined by EXAFS fitting of the Pt L_3-edge on reduced and spent catalysts after APR of ethylene glycol in the range 3 < k < 12 $Å^{-2}$, 1.1< R< 4 Å. Fitting is performed using the Pt, PtO, PtO_2, PtN, PtS and PPt crystal structures [10].

Catalyst	Shell	E_0 (eV)	CN	R (Å)	σ^2 (Å)	R-factor
Pt/CNF-HT reduced	Pt-Pt	6.03	8.84	2.74	0.0085	0.050
	Pt-O		0.43	1.75	0.0127	
	Pt-Pt		2.91	3.89	0.0081	
Pt/CNF-HT spent	Pt-Pt	6.85	9.87	2.75	0.0074	0.050
	Pt-O		0.49	1.74	0.0091	
	Pt-Pt		1.45	3.89	0.0029	
Pt/N-CNF reduced	Pt-Pt	3.56	7.78	2.72	0.0142	0.050
	Pt-N		1.03	1.97	0.0107	
	Pt-Pt		0.60	3.90	0.0038	
Pt/N-CNF spent	Pt-Pt	6.53	8.92	2.75	0.0087	0.049
	Pt-N/O		0.85	1.88	0.0139	
	Pt-Pt		3.11	3.90	0.0102	
Pt/S-CNF reduced	Pt-Pt	5.77	3.92	2.72	0.0087	0.040
	Pt-S		1.29	2.31	0.0019	
	Pt-O		2.10	1.95	0.0248	
Pt/S-CNF spent	Pt-Pt	4.11	5.92	2.72	0.0096	0.044
	Pt-S		1.15	2.30	0.0011	
	Pt-O		1.69	1.91	0.0244	
Pt/P-CNF Reduced*	Pt-Pt	6.26	9.33	2.75	0.0064	0.005
	Pt-P		0.70	2.27	0.0079	
	Pt-Pt		1.51	3.90	0.0009	
Pt/P-CNF spent*	Pt-Pt	7.79	11.63	2.76	0.0058	0.015
	Pt-P		0.48	2.24	0.0175	
	Pt-Pt		3.64	3.88	0.00718	
Pt/B-CNF Reduced*	Pt-Pt	7.85	10.28	2.75	0.0057	0.005
	Pt-O		0.07	1.91	0.0151	
	Pt-Pt		2.52	3.90	0.0028	
Pt/B-CNF spent*	Pt-Pt	8.14	10.94	2.76	0.0056	0.006
	Pt-O		0.24	1.86	0.0120	
	Pt-Pt		2.81	3.89	0.0029	

* Spectra fitted in R-space.
Accuracy coordination number, CN: ± 10-20%. Interatomic distance R: ± 0.02Å.

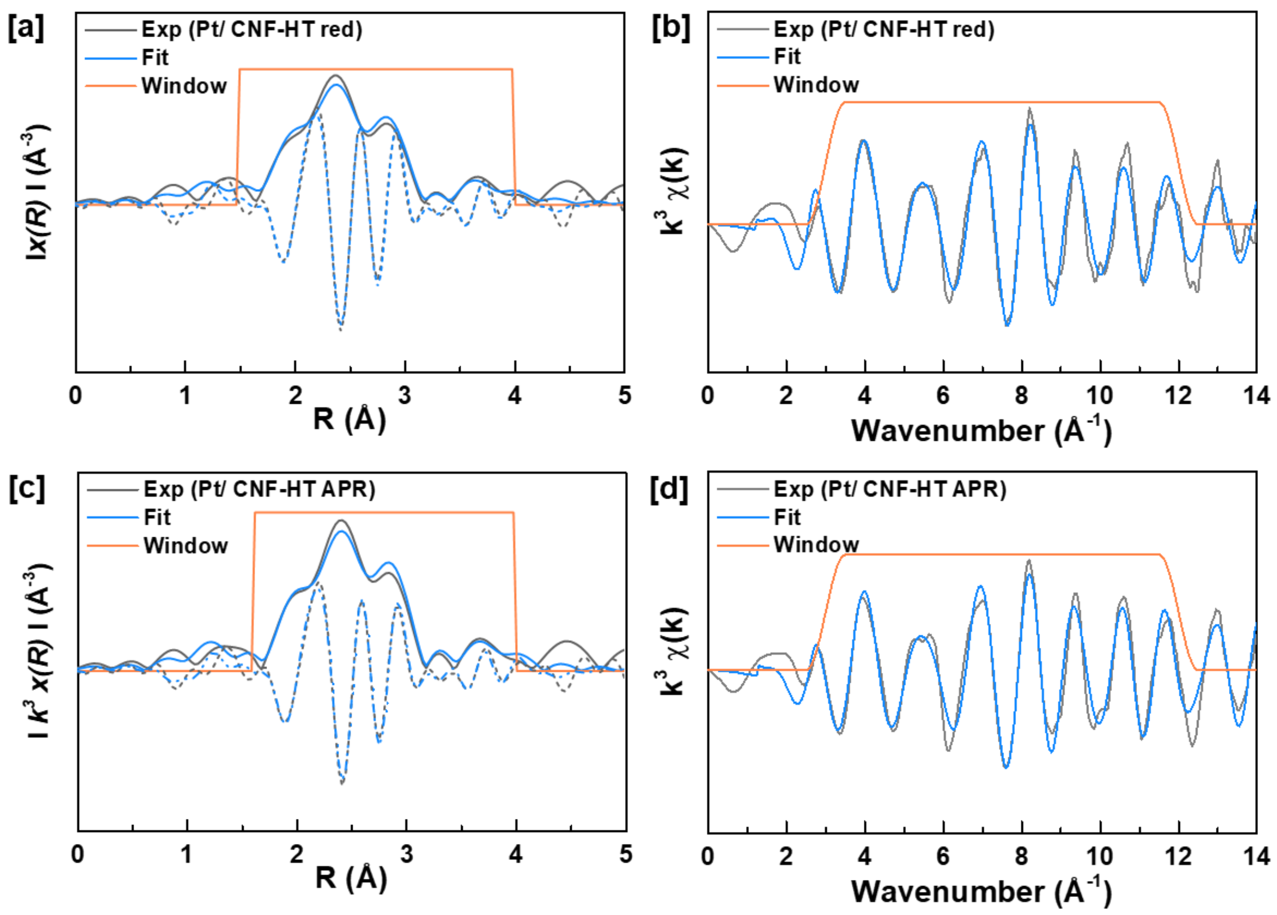

Figure S 20. Pt L_3-edge EXAFS fitting curves of Pt/CNF-HT after catalyst reduction [a] in R space [b] in k space. After APR of ethylene glycol at 225°C and 30 bars (WHSV = $9h^{-1}$) [c] in R space [d] in k space.

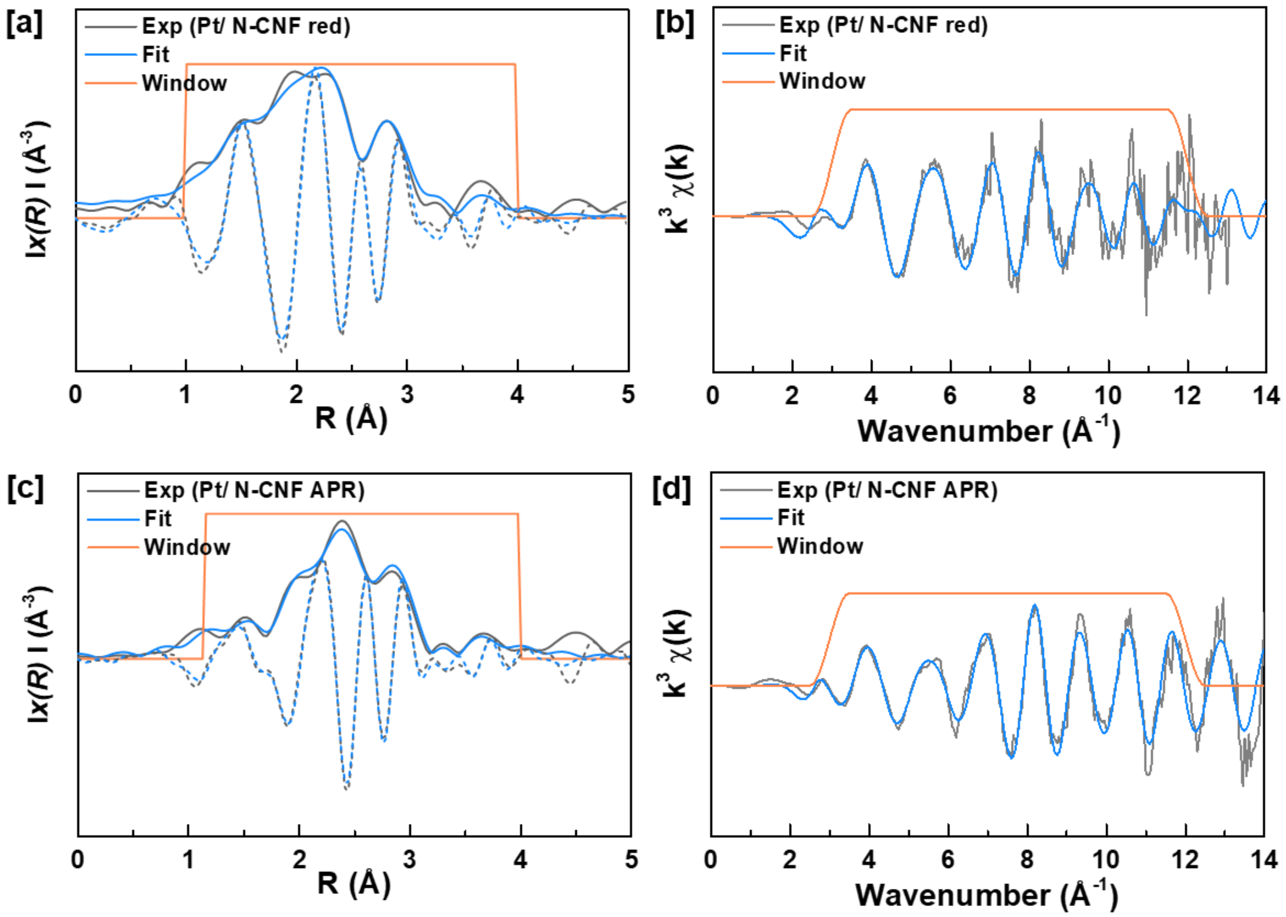

Figure S 21. Pt L_3-edge EXAFS fitting curves of Pt/N-CNF data after catalyst reduction [a] in R space [b] in k space. After APR of ethylene glycol at 225°C and 30 bars (WHSV = $9h^{-1}$) [c] in R space [d] in k space.

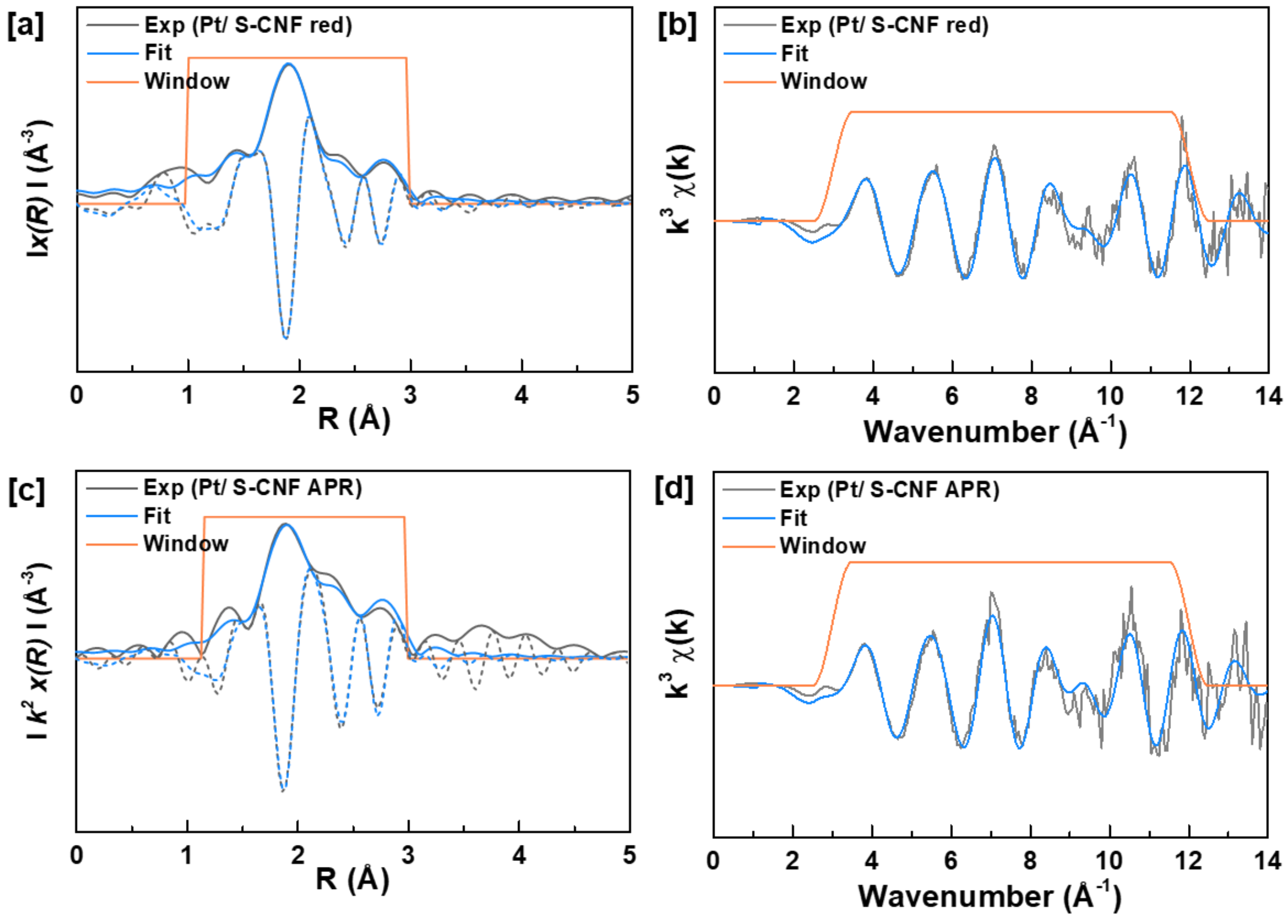

Figure S 22. Pt L_3-edge EXAFS fitting curves of Pt/S-CNF data after catalyst reduction [a] in R space [b] in k space. After APR of ethylene glycol at 225°C and 30 bars (WHSV = $9h^{-1}$) [c] in R space [d] in k space.

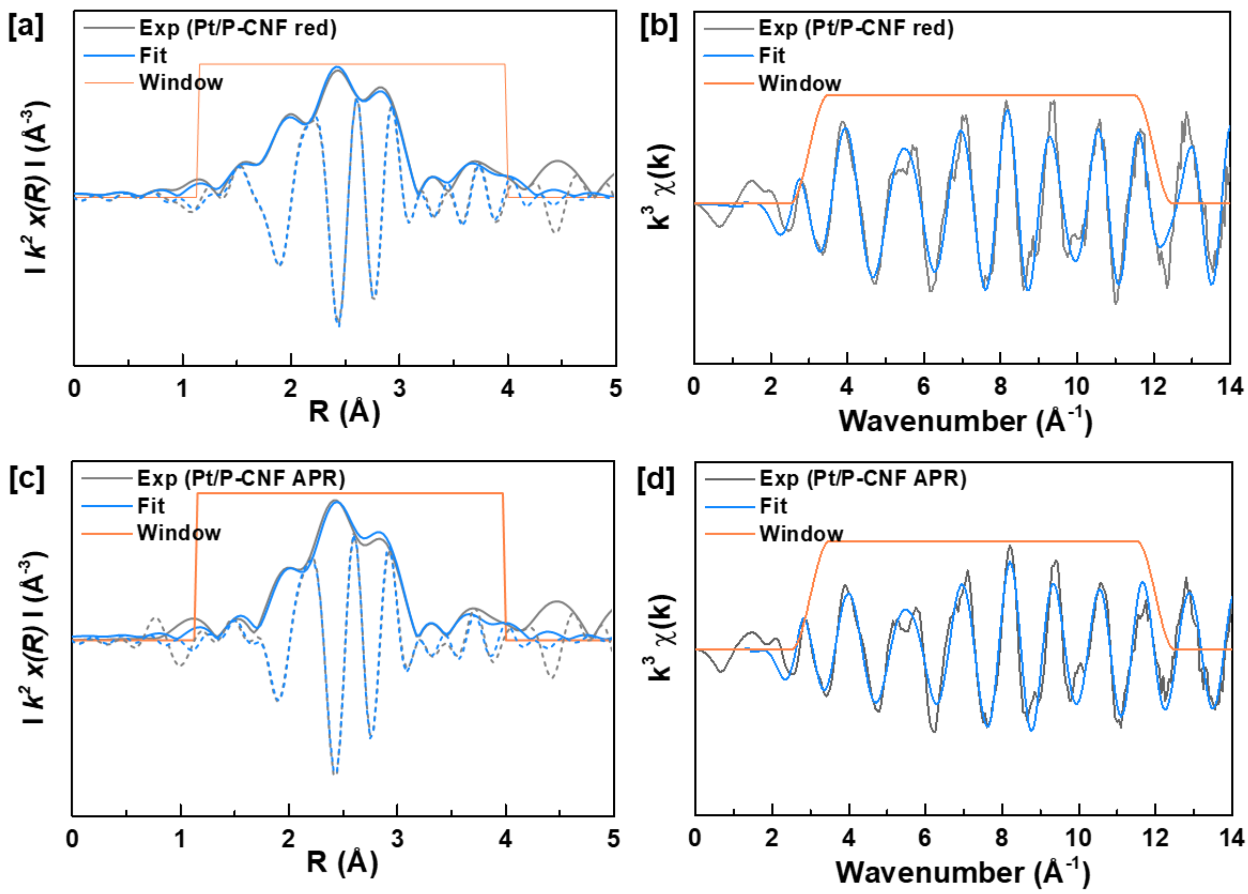

Figure S 23. Pt L_3-edge EXAFS fitting curves of Pt/P-CNF data after catalyst reduction [a] in R space [b] in k space. After APR of ethylene glycol at 225°C and 30 bars (WHSV = $9h^{-1}$) [c] in R space [d] in k space.

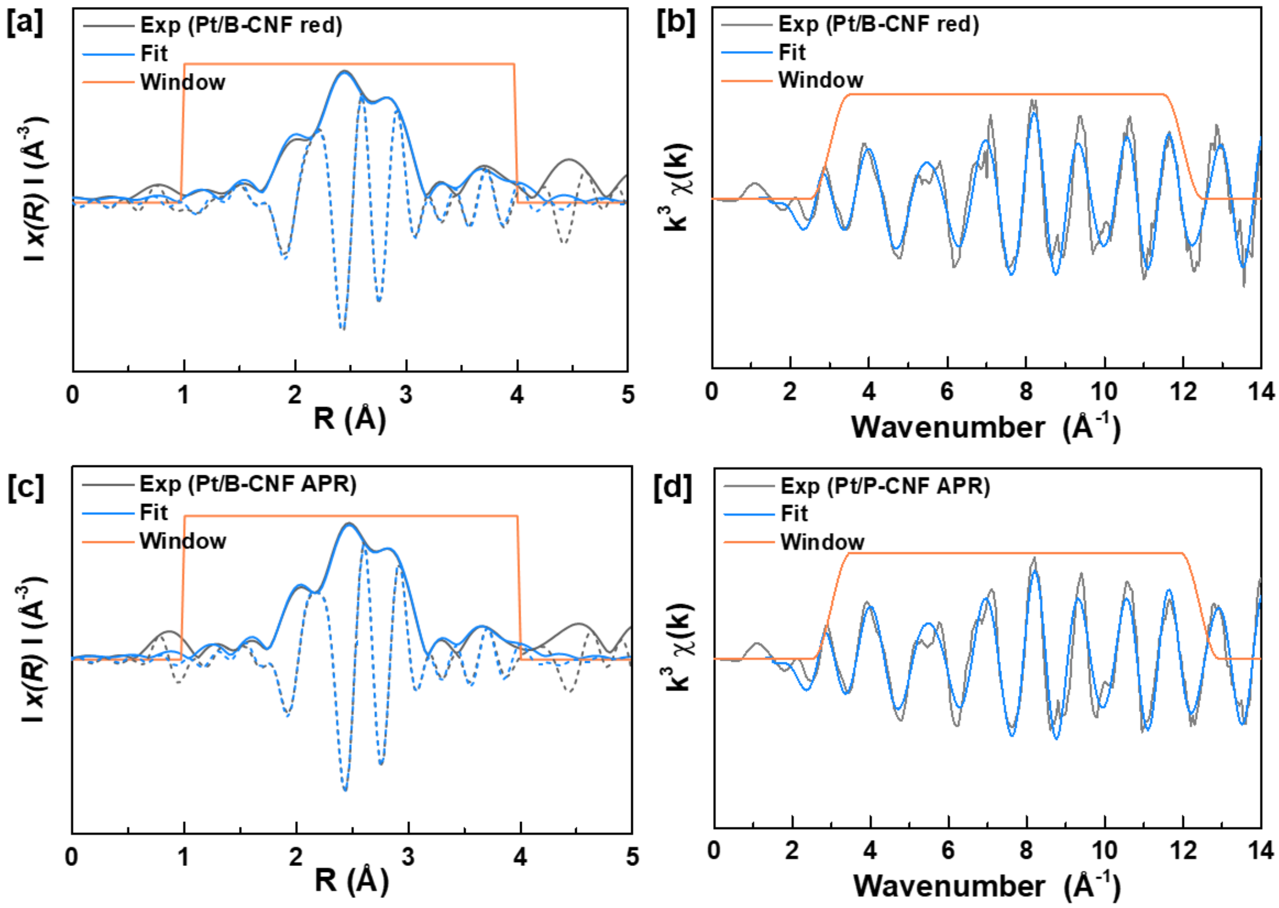

Figure S 24. Pt L_3-edge EXAFS fitting curves of Pt/B-CNF data after catalyst reduction [a] in R space [b] in k space. After APR of ethylene glycol at 225°C and 30 bars (WHSV = $9h^{-1}$) [c] in R space [d] in k space.

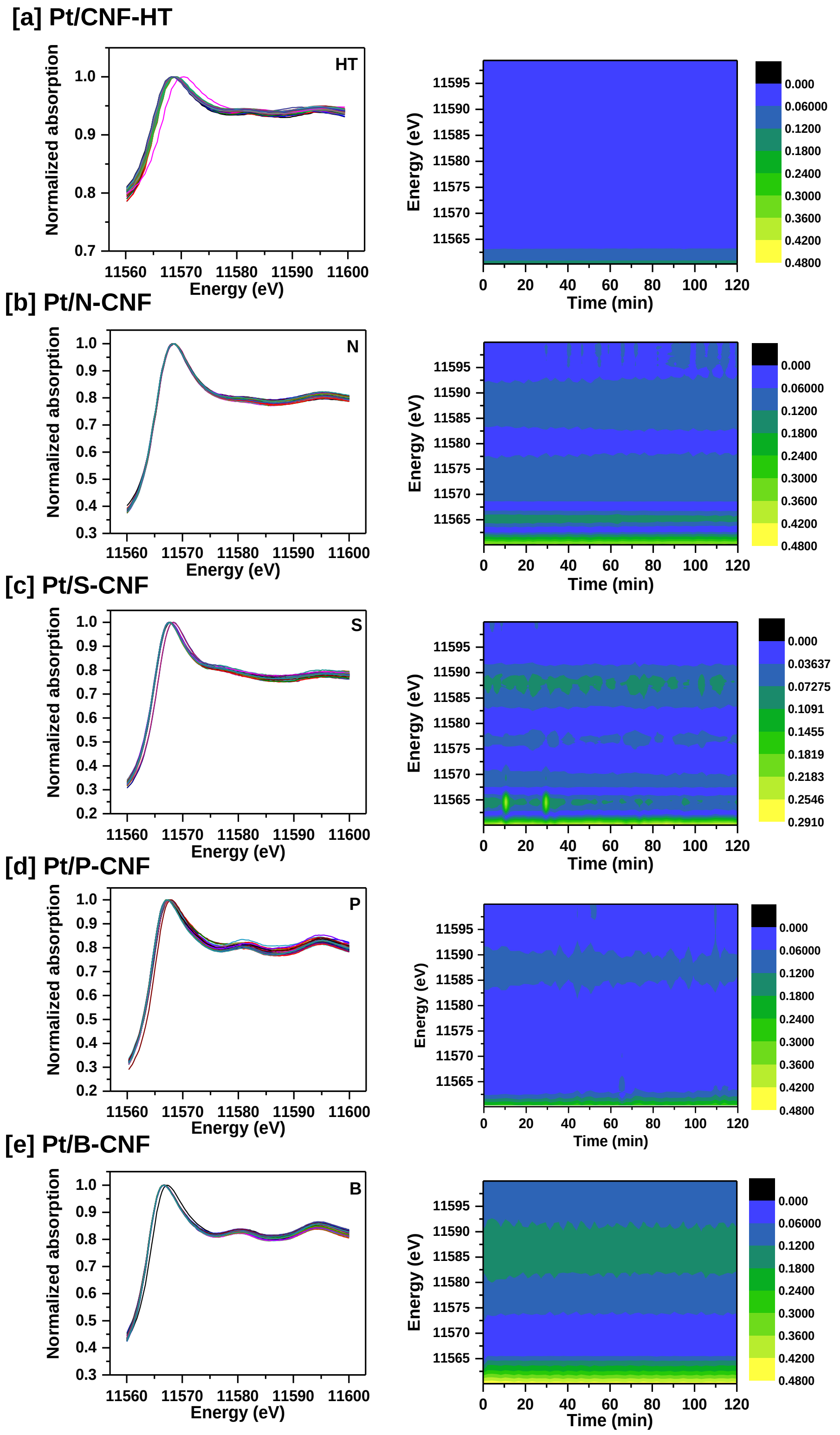

Figure S 25. Normalized XANES spectra at the Pt L_3-edge acquired during APR of ethylene glycol at 225°C and 30bar. The spectra were adjusted to the range available from the standards obtained from the Materials Project, 11.56-11.6 keV [9]. Minimal error of the contribution plot (%) is included. Each sample has a percentage scale from black (low error) to bright yellow (high error).

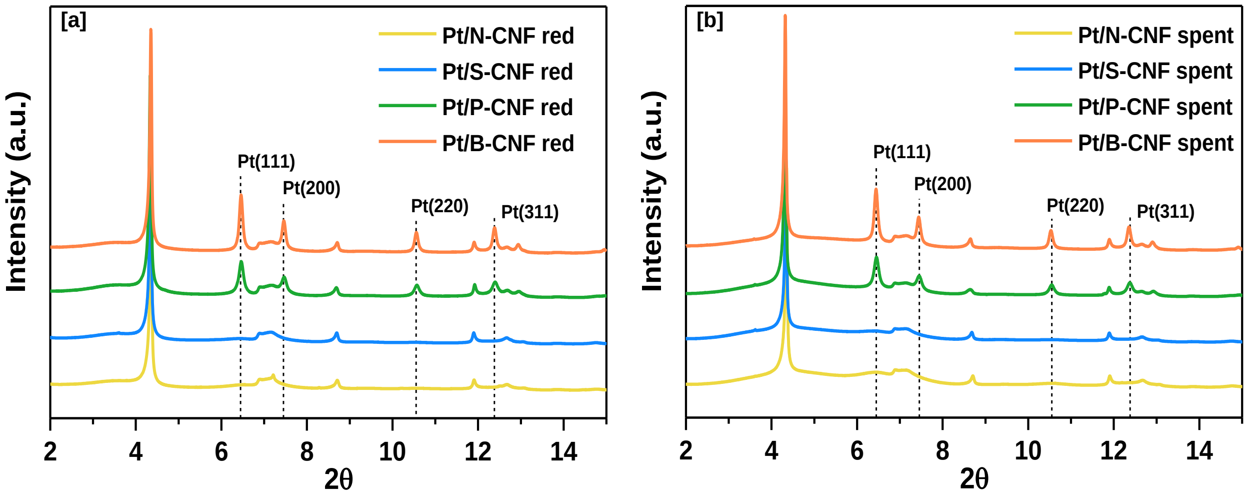

Figure S 26. XRD patterns (λ = 0.255 Å) recorded during in situ aqueous phase reforming of ethylene glycol. [a] Reduced catalysts [b] Spent catalysts

REFERENCES

[1] K. Friedel Ortega, R. Arrigo, B. Frank, R. Schlögl, A. Trunschke, Chem. Mater. 28 (2016) 6826–6839.

[2] X. Cui, Z. Pan, L. Zhang, H. Peng, G. Zheng, Adv. Energy Mater. 7 (2017) 1701456.

[3] J.F. Moulder, J. Chastain, Handbook of X-Ray Photoelectron Spectroscopy: A Reference Book of Standard Spectra for Identification and Interpretation of XPS Data, Physical Electronics Division, Perkin-Elmer Corporation, 1992.

[4] A.P. Terzyk, Colloids Surf. Physicochem. Eng. Asp. 177 (2001) 23–45.

[5] J. Wu, C. Jin, Z. Yang, J. Tian, R. Yang, Carbon 82 (2015) 562–571.

[6] W.E. Morgan, W.J. Stec, R.G. Albridge, J.R. Van Wazer, Inorg. Chem. 10 (1971) 926–930.

[7] S.C. Lyu, J.H. Han, K.W. Shin, J.H. Sok, Carbon 49 (2011) 1532–1541.

[8] S. Jacques, A. Guette, X. Bourrat, F. Langlais, C. Guimon, C. Labrugere, Carbon 34 (1996) 1135–1143.

[9] F. Herold, T. Imhof, P. Roumeliotis, P. Schühle, M. Ledendecker, M. Rønning, Carbon 207 (2023) 207–218.

[10] A. Jain, S.P. Ong, G. Hautier, W. Chen, W.D. Richards, S. Dacek, S. Cholia, D. Gunter, D. Skinner, G. Ceder, K.A. Persson, APL Mater. 1 (2013) 011002.
